# Engineering charge transfer by tethering halogens to covalent organic frameworks for photocatalytic sacrificial hydrogen evolution†

**DOI:** 10.1039/d5sc00082c

**Published:** 2025-06-06

**Authors:** Fangpei Ma, Xiao Chi, Ying Wen, Qihong Yue, Tao Chen, Xiaojiang Yu, Xiaoling Liu, Yu Zhou, Jun Wang

**Affiliations:** a State Key Laboratory of Materials-Oriented Chemical Engineering, College of Chemical Engineering, Nanjing Tech University Nanjing 211816 China njutzhouyu@njtech.edu.cn junwang@njtech.edu.cn; b Department of Physics, National University of Singapore 117576 Singapore; c Singapore Synchrotron Light Source, National University of Singapore 5 Research Link 117603 Singapore

## Abstract

Covalent organic frameworks (COFs) are promising versatile organic semiconductors for the photocatalytic hydrogen evolution reaction (HER) but usually rely on the utilization of expensive Pt species as the co-catalyst and exhibit inferior activity in seawater relative to that in pure water. Herein, different halogen atoms (F, Cl, and Br) were integrated into a COF to optimize the photochemical properties, carrier thermodynamics, and kinetics. The Cl-containing COF TpPaCl exhibited effective co-catalyst-free HER performance under visible light irradiation, with a high HER rate of 16.3 mmol g^−1^ h^−1^ in artificial seawater (11.5 mmol g^−1^ h^−1^ in real seawater), outperforming that in pure water. The remarkable performance came from the fine and efficient adjustment of the local environment of the COF by the electronegative halogen atoms. Theoretical calculations and soft X-ray absorption spectroscopy (XAS) indicated that the carbon atoms adjacent to the halogen group exhibited strong interaction towards the key intermediate (H*) in the HER process and served as photoactive H_2_ evolution sites. The strong polarization derived from the halogen also greatly improved the formation of long-life electrons and accelerated the successive charge transfer. The kinetics were enhanced after salt adsorption in seawater, contributing to the superior HER rate in seawater.

## Abstract 2

Halogen-containing COFs achieve effective HER performance under visible light irradiation. The strong polarization derived from the halogen greatly improves the formation of long-life electrons and accelerates the successive charge transfer.

## Graphical abstract

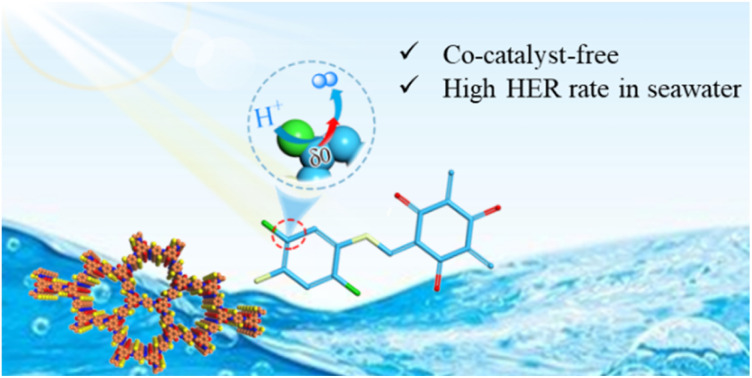

## Introduction

The global energy crisis and climate problem convey the urgency to search for clean and renewable energy sources to alleviate the emission of greenhouse gases and the consumption of traditional fossil fuels.^1–4^ Hydrogen (H_2_) is considered to be the most promising non-carbon energy source, with high energy density and the release of only water. Solar-driven photocatalytic water splitting is a highly attractive sustainable strategy to produce H_2_, with the advantage of converting inexhaustible solar energy into chemical substances.^1–3^ However, the efficiency of the photocatalytic H_2_ evolution reaction (HER) is limited due to the unfavorable thermodynamics and kinetics.^5–7^ For promoting the HER efficiency, Pt species are frequently loaded on photocatalysts, especially organic semiconductors, to enhance the carrier thermodynamics and kinetics, attributable to the most suitable working function, but their high price greatly hinder large-scale applications.^8–10^ Furthermore, the HER in seawater is more preferred compared with the one in pure water, because seawater is the largest water reservoir on Earth while clean water is also a precious resource. Nonetheless, the majority of photocatalysts exhibited inferior HER activity in seawater relative to that in pure water, even in the presence of the Pt co-catalyst.^7,9,11^ A co-catalyst-free system is challenging and still rare for the photocatalytic HER process as yet, especially for the one in the seawater.

Covalent organic frameworks (COFs) have exhibited great potential serving as organic semiconductors in the photocatalysis field,^12,13^ due to their rich pore structure, versatile chemical composition, and molecular level tunability and predictivity. To improve the HER performance, sulfone,^14^ triazine,^15^ and benzothiadiazole^16^ moieties have been integrated into the COF to adjust the light-harvesting ability, redox potential, and charge separation and transfer behavior. Meanwhile, functional units like bipyridine and thiophene on the COFs enable the simultaneous occurrence of hydrogen evolution and oxygen evolution, promoting the progress of the overall water splitting reaction.^17–19^ Most of these COF-based photocatalysts were active in the presence of Pt co-catalysts and produced negligible H_2_ without a co-catalyst in many cases.^20,21^ A significant reason can be assigned to the weak interaction between the organic skeleton and the H adsorption intermediates (H*), which hinders the H_2_ formation and desorption processes. Considering the full designability of the COF architecture, it is rational that the molecular engineering of the building blocks provides a favorable opportunity to regulate the carrier thermodynamics and kinetics of COFs to allow the straightforward photocatalytic H_2_ production without the assistance of a co-catalyst. Although several sporadic examples have been reported,^13,20^ effective COF-derived co-catalyst-free photocatalytic HER activity is to be developed, and none of them has been explored for the HER in seawater.

Herein, we have demonstrated the feasibility of halogen functionalization to optimize the carrier thermodynamics and kinetics for the visible-light-driven HER in seawater under co-catalyst free conditions. The target halogen functionalized COFs and non-halogen control sample were synthesized through Schiff base condensation between 1,3,5-triformylphloroglucinol (Tp) and diamino-containing monomers with different halogens (F, Cl, and Br) or without any halogen. The strong electronegativity of the halogen atom finely adjusts the local environment of the COF to modulate the photochemical properties, carrier thermodynamics, and kinetics. In particular, the integration of the Cl atom significantly reduces the energy barrier of the H* adsorption on the adjacent carbon atoms that serve as the photoactive sites for H–H bond formation and H_2_ desorption. As a result, the Cl-tethered catalyst TpPaCl is highly active in the HER in the absence of any co-catalyst under visible-light irradiation. The adsorption of certain salt in seawater further optimizes the carrier behavior, achieving a high HER rate of 16.3 mmol g^−1^ h^−1^ in artificial seawater (11.5 mmol g^−1^ h^−1^ in real seawater). Theoretical calculations, soft X-ray absorption spectroscopy (XAS), transient absorption spectroscopy, and quasi *in situ* ESR characterization were conducted to identify the H_2_ evolution sites and unravel the role of halogen in lowering the thermodynamic barrier for H_2_ evolution and accelerating the HER kinetics.

## Experimental section

### Materials

All the chemicals and reagents were commercially available and used without additional purification. 1,3,5-Triformylphloroglucinol (Tp, 99%) and 2,5-dibromobenzene-1,4-diamine (PaBr, 97%) were purchased from Shanghai Tensus Biotech Co., Ltd. 2,5-Difluorophenylene-1,4-diamine (PaF, 95%) was purchased from Bidepharm Co., Ltd. 2,5-Dichlorobenzene-1,4-diamine (PaCl, 98%), *n*-butanol (99.8%), and tetrahydrofuran (THF, 99.9%) were purchased from Aladdin Industrial Corporation. Mesitylene (99%) was purchased from Energy Chemical. Dioxane (99%) was purchased from Jiangsu Yonghua Fine Chemical Co., Ltd. 1,4-Benzenediamine (Pa, 99%) and *o*-dichlorobenzene (2.5 mL, 99%) were purchased from Macklin Co. Ltd. Acetic acid (99%) was purchased from Shanghai Shenbo Chemical Co., Ltd. Ethanol (99.8%) was purchased from Sinopharm Chemical Reagent Co., Ltd.

### Catalyst preparation

#### Synthesis of TpPaF

A microwave tube (70 mL) was charged with Tp (63 mg, 0.3 mmol), PaF (64.8 mg, 0.45 mmol), *n*-butanol (2.5 mL), *o*-dichlorobenzene (2.5 mL, 99%), and acetic acid (6 M, 1 mL). The mixture was heated at 120 °C for 1 h under microwave irradiation in a CEM MARS6 microwave reactor. After that, the red precipitates were washed with ethanol (3 × 5 mL) and THF (3 × 5 mL), which were collected by filtration and Soxhlet extracted with THF for 24 h. After drying at 120 °C under vacuum for 12 h, the product was obtained as a brown powder (yield: 81%).

#### Synthesis of TpPaCl

TpPaCl was synthesized with the same procedure as TpPaF by using the initial gel composition of Tp (63 mg, 0.3 mmol), PaCl (79.7 mg, 0.45 mmol), *n*-butanol (2.5 mL), *o*-dichlorobenzene (2.5 mL) and acetic acid (6 M, 1 mL) (yield: 91%).

#### Synthesis of TpPaBr

TpPaBr was synthesized using the same procedure as TpPaF with the initial gel composition of Tp (63 mg, 0.3 mmol), PaBr (119.7 mg, 0.45 mmol), *n*-butanol (2.5 mL), *o*-dichlorobenzene (2.5 mL), and acetic acid (6 M, 1 mL) (yield: 86%).

#### Synthesis of TpPaH

TpPaH was synthesized using the same procedure as TpPaF with the initial gel composition of Tp (63 mg, 0.3 mmol), 1,4-benzenediamine (Pa, 32.4 mg, 0.45 mmol), *n*-butanol (2.5 mL), *o*-dichlorobenzene (2.5 mL), and acetic acid (6 M, 1 mL) (yield: 82%).

### Characterization studies

A series of characterization studies were conducted, including powder X-ray diffraction (PXRD), scanning electron microscopy (SEM), transmission electron microscopy (TEM), X-ray photoelectron spectroscopy (XPS), Fourier transform infrared (FT-IR) spectroscopy, elemental analysis, thermogravimetric analysis (TGA), solid-state ^13^C cross-polarization/magic angle spinning nuclear magnetic resonance (CP/MAS NMR) spectroscopy, electron paramagnetic resonance (ESR) spectroscopy, transient absorption spectroscopy (TAS), and soft X-ray absorption spectroscopy (XAS). Photocurrent measurements and electrochemical impedance spectroscopy (EIS) were conducted by using a CHI 760E electrochemical workstation (Shanghai Chenhua, China). Details are provided in the ESI.†

### Photocatalytic hydrogen evolution

The catalyst was dispersed in 20 mL of H_2_O containing 0.1 M ascorbic acid, and the reactor was then purged with Ar flow for 15 min to remove air. The reaction proceeded at room temperature under irradiation by using a 300 W Xe lamp (PLS-SME300E H1, Xenon Lamp Source, Beijing PerfectlightTechnology Co., Ltd.) equipped with a 420 nm cutoff filter. Photocatalytic seawater decomposition was conducted using the same procedure by replacing the same amount of pure water with artificial seawater (26.518 g per L NaCl, 3.305 g per L MgSO_4_, 2.447 g per L MgCl_2_, 1.141 g per L CaCl_2_, 0.725 g per L KCl, 0.202 g per L NaHCO_3_, and 0.083 g per L NaBr) and real seawater sampled from Qingdao, China, specifically from the waters of Lingshan Bay in Qingdao. Parallel investigation was also conducted in the presence of hexachloroplatinic acid (2 wt% Pt to photocatalyst). Gas products were analyzed by using a gas chromatograph (GC-9860-5CNJ, Nanjing Hope Analytical Equipment Co., Ltd, China) configured with a thermal conductivity detector (TCD) and a flame ionization detector (FID). After the reaction, the spent sample was collected, washed with water, dried at 120 °C, and then charged into the next to evaluate the recycling performance. The apparent quantum yield (AQY) was measured at different wavelengths (420 nm, 450 nm, 500 nm, 550 nm, 600 nm, and 650 nm) using cut-on filters of monochromatic light.

### Theoretical study

Density functional theory (DFT) calculations were carried out using the Gaussian 09 program package. The structural models for the corresponding representative fragments of COFs were optimized with the B3LYP/def2-SVP level of theory. We calculated the ionization potential (IP), electron affinity (EA), exciton ionization potential (IP*), and exciton electron affinity (EA*) of COFs plus the Gibbs free energy change for H* adsorption (Δ*G*_H*_) on these COFs. Details are provided in the ESI.†

## Results and discussion

### Formation of halogenated COFs

**Fig. 1:**
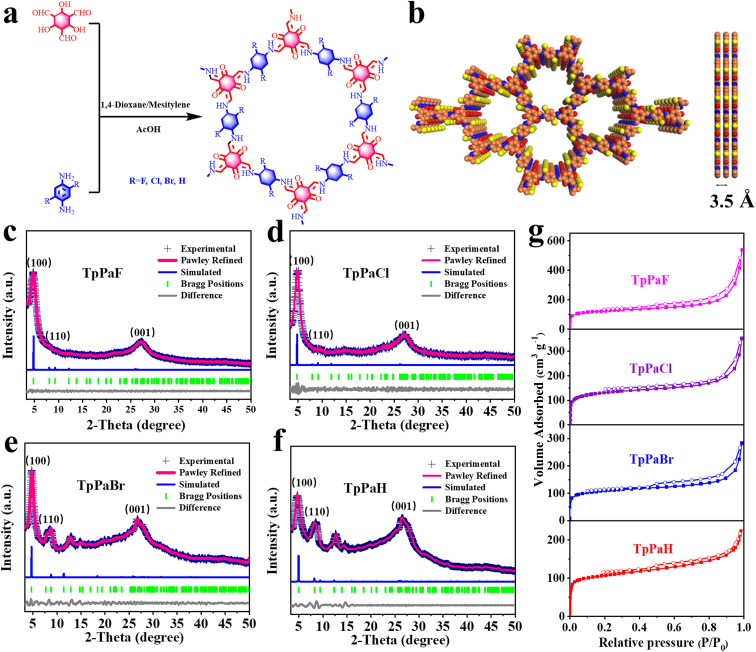
(a) Schematic illustration of the synthesis of TpPaX (X = F, Cl, Br, H). (b) Lattice structure in top and side views (orange: carbon; blue: nitrogen; red: oxygen; yellow: chlorine) for the AA-stacking model of TpPaCl. (c–f) Experimentally observed (ink blue) and Pawley refined (magenta) PXRD patterns with their refinement difference (gray), the reflections, and the simulated ones (blue) for the eclipsed AA-stacking model. (g) N_2_ sorption isotherms of TpPaX.

Fig. 1a shows the synthesis of TpPaX (X = F, Cl, Br, H) through condensation of Tp, respectively, with 2,5-difluoro-1,4-benzenediamine, 2,5-dichlorobenzene-1,4-diamine, 2,5-dibromobenzene-1,4-diamine, and 1,4-phenylenediamine in a microwave-assisted solvothermal route by using acetic acid (AcOH) as the catalyst and a *n*-butanol/*o*-dichlorobenzene mixture as the solvent. No residue noble metals were detectable by high-resolution inductively coupled plasma mass spectrometry (ICP-MS) (Table S1†). The PXRD patterns of TpPaX showed multiple peaks for the (100), (110), and (001) planes (Fig. 1c–f and S1–S4†), revealing the crystal structure.^22,23^ The comparison of experimental and simulated PXRD patterns (Fig. 1b and S1–S4†) shows the matching of their structure with the AA stacking and the P6/M spacing group. By contrast, the simulated AB stacking pattern was far from the experimental PXRD pattern. The Pawley refined PXRD profiles reproduced the experimental ones of TpPaF (*R*_wp_ = 1.96% and *R*_p_ = 1.49%), TpPaCl (*R*_wp_ = 4.81% and *R*_p_ = 3.72%), TpPaBr (*R*_wp_ = 2.06% and *R*_p_ = 1.67%) and TpPaH (*R*_wp_ = 3.48% and *R*_p_ = 2.35%), with the following unit cell parameters of *a* = *b* = 22.86 Å for TpPaF, *a* = *b* = 22.95 Å for TpPaCl, *a* = *b* = 25.26 Å for TpPaBr, and *a* = *b* = 22.76 Å for TpPaH (Tables S2–S5†).

FT-IR spectra (Fig. S5–S8†) demonstrated the disappearance of the band from N–H stretching vibration of diamine (3180–3400 cm^−1^) and the attenuation of C=O stretching vibration from Tp (1639 cm^−1^) over TpPaX. The characteristic C–N stretching vibration at about 1241 cm^−1^ revealed the condensation of aldehyde and ammonia.^24^ The observation of the characteristic band at 1567 cm^−1^ for the C=C bond indicated the formation of a ketone structure.^25^ The chemical structure of TpPaX was further confirmed by solid-state ^13^C CP/MAS NMR spectra (Fig. S9–S12†). The surrounding peaks of 146.6 ppm and 107.5 ppm were attributed to the connection of carbon–nitrogen bonds (C–N) and carb.^26^ The elemental analysis showed that the C/N ratio of each TpPaX was close to the theoretical value (Table S6†). TGA indicated that TpPaX was stable up to ∼330 °C in N_2_ (Fig. S13–S16†), revealing high thermal stability.

SEM images (Fig. S17†) demonstrated the morphology of TpPaX. TpPaCl had a slender fiber shape with a width of ∼100 nm and a length at the micrometer level. TpPaF exhibited the morphology of flake foam composed of small particles with the size of ten nanometers. TpPaBr showed a curved ultra-flaky shape with a thickness of 10 nm. The flower-like particles of TpPaH came from the aggregation of the spike-like petals with a length of about 2 μm. The crystal structure of the typical sample TpPaCl was further visualized by TEM images (Fig. S18†), showing the existence of the (001) facet with an interlayer distance of ∼0.33 nm.^27^ XPS analysis was performed to study the surface chemical composition and electronic state of TpPaX (Fig. S19–S22†). Taking TpPaCl as an example, the survey scan XPS spectrum (Fig. S19a†) illustrated the peaks at 284.9, 400.6, 528.8, and 197.8 eV for C 1s, N 1s, O 1s, and Cl 2p signals, respectively. The high-resolution C 1s spectrum of TpPaCl (Fig. S19b†) was deconvolved into four peaks of 284.9, 286, 286.7, and 288.8 eV, corresponding to the sp^2^-hybrid carbon (C–C), aldehyde amine condensation carbon (C–N), halogen carbon (C–Cl), and carbon in the carbonyl group (C=O), respectively. In the high-resolution N 1s XPS spectrum, only one peak of 400.6 eV was observed, attributable to the C–N of TpPaCl (Fig. S19c†).^28^ The characteristic Cl 2p signals were observed at 201.2 and 202.7 eV (Fig. S19d†). The F 1s signals at 687.9 and 689.7 eV and Br 3d signals at 70.1 and 71.8 eV were, respectively, observed in the high-resolution F 1s XPS spectrum of TpPaF and Br 3d XPS spectrum of TpPaBr (Fig. S20 and S22†).^29^

Nitrogen sorption isotherms of TpPaX (Fig. 1g) were of type IV, with a steep uptake at the low relative pressure from the micropores and weak uptake at higher pressure from the formation of certain mesopores due to the particle packing.^16^ The surface areas of TpPaX were 404 to 493 m^2^ g^−1^, which were associated with the pore volume of 0.34 to 0.83 cm^3^ g^−1^. The pore size distribution curves revealed the most probable pore size to be 1–2 nm, visualizing the presence of abundant micropores (Fig. S23†), which was further reflected by a high proportion of micropores in these materials (Table S7†). Water vapor adsorption isotherms of TpPaX measured at 298 K (Fig. S24†) were of type II, with a higher adsorption capacity of 49 wt% for TpPaCl than those for TpPaBr (25 wt%), TpPaF (22 wt%) and TpPaH (19 wt%). This phenomenon suggests a better hydrophilic property of TpPaCl due to the existence of polar atoms (Cl, N, and O) and a high surface area.^30,31^

### Photoelectrochemical properties

**Fig. 2:**
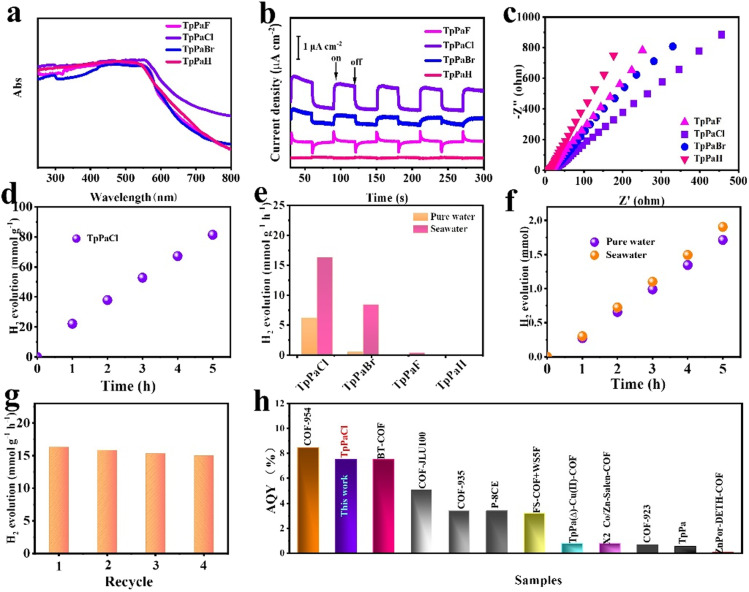
(a) UV-vis DRS spectra, (b) transient photocurrent responses, and (c) electrochemical impedance spectra of TpPaX (X = F, Cl, Br, and H). (d) Time-resolved HER over TpPaCl in seawater under visible light irradiation. (e) HER rates of TpPaX in pure water and seawater. (f) Time-resolved HER of TpPaCl in pure water and seawater. (g) Recycling HER of TpPaCl in seawater. (h) Comparison of AQY of TpPaCl with previous state-of-the-art photocatalysts (details are provided in Tables S8 and S9†). Reaction conditions: 2 mg COFs, *λ* > 420 nm, 0.1 M ascorbic acid, room temperature, and 5 h. In particular, 5 mg TpPaCl and hexachloroplatinic acid aqueous solution (2 wt% Pt to photocatalyst) were added to the system for (f).

UV-vis spectra revealed the strong absorption of TpPaX from UV to visible-light range, and TpPaCl exhibited the best light harvesting ability with extended absorption relative to the other three samples (Fig. 2a).^32^ Transient photocurrent measurements were used to study the charge separation and migration behavior. Each TpPaX showed a transient response under visible-light illumination, and the photocurrent intensity was in the sequence of TpPaCl > TpPaBr > TpPaF > TpPaH (Fig. 2b). The highest light response of TpPaCl reflected the most favorable light harvesting, excitation, charge separation, and transport efficiency.^33,34^ The electrochemical impedance spectroscopy (EIS) test indicated that TpPaCl also exhibited the lowest resistance, as reflected by the smallest semicircle diameter in the Nyquist diagram (Fig. 2c), facilitating the reduction of the charge transfer barrier.^35,36^

Steady-state photoluminescence (PL) spectra of TpPaX (Fig. S25†) demonstrated the decrease of the peak intensity in the order of TpPaH > TpPaF > TpPaBr > TpPaCl, reflecting the gradual enhancing ability to inhibit the radiative charge recombination. Time-resolved PL decay spectra were collected to estimate the solid-state excited state lifetime associated with the kinetics of charge transport. As shown in Fig. S26.† TpPaCl showed the longest emission lifetime (*τ*_avg_ = 15.5 ns), superior to those of TpPaBr (13.0 ns), TpPaF (3.4 ns), and TpPaH (1.6 ns) (Table S8†). Therefore, TpPaCl has the most favorable efficiency for the excited state charge separation, which in turn will facilitate the successive participation of the excited electrons and holes in the photo-redox process.^24^ All these results revealed the feasibility of modulating the photoelectrochemical properties of COFs by finely adjusting the building blocks *via* molecular engineering.

### Photocatalytic hydrogen evolution

TpPaX series were engaged in H_2_ production under visible-light irradiation by using pure water and artificial seawater (for simplification, the term seawater is used to denote artificial seawater in the following). The initial evaluation was conducted under co-catalyst-free conditions. Among them, TpPaCl exhibited the best performance, leading to continuous H_2_ evolution by using pure water and seawater. The HER rate of TpPaCl was as high as 6.2 mmol g^−1^ h^−1^ in pure water (Fig. 2d and e) and significantly increased to 16.3 mmol g^−1^ h^−1^ when switching to seawater. The HER rate in seawater is the highest one so far in the co-catalyst free systems (Table S8†) and is even comparable to various state-of-the-art photocatalysts in the presence of Pt species as the co-catalyst (Table S9†). The molecular structure was found to significantly affect the HER rate. TpPaBr was also active in the photocatalytic HER under identical conditions, giving the HER rates of 0.5 and 8.4 mmol g^−1^ h^−1^, respectively, in pure water and seawater. By contrast, TpPaF caused only weak activity, with the HER rate of 0.35 mmol g^−1^ h^−1^, while TpPaH was almost inert in the reaction by using either pure water or seawater. The measurement of the apparent quantum yield (AQY) of TpPaCl under monochromatic light (Fig. S27†) showed an AQE of 2.12% at 420 nm by using seawater in the absence of any co-catalyst. The cyclic H_2_ evolution reaction of TpPaCl was assessed in seawater, and a relatively stable HER rate was achieved in the four cycles, illustrating the good recyclability (Fig. 2g). The influence of different salts on the TpPaCl catalyzed H_2_ production was investigated under visible-light irradiation (Fig. S28†). The result indicated that the presentation of each salt alone promoted the photocatalytic HER rate, and the most conspicuous promotion was observed by using NaCl, implying that NaCl plays a vital role in accelerating the HER in seawater. The HER rate of TpPaCl was 11.5 mmol g^−1^ h^−1^ by using the real seawater (Fig. S29†), which is still higher than that by using pure water while slightly lower than that using artificial seawater. This result confirmed the enhanced HER activity in the seawater relative to the pure water. Photocatalytic HER activity of TpPaX was further investigated in pure water by using Pt species as the co-catalyst (Fig. S30–S32†). The average HER rate of TpPaCl reached 68.56 mmol g^−1^ h^−1^ (5 mg catalyst), nearly 33 times faster than that of TpPaH (2.1 mmol g^−1^ h^−1^) and also superior to those of TpPaBr (27.62 mmol g^−1^ h^−1^) and TpPaF (6.3 mmol g^−1^ h^−1^). The constant HER rate of TpPaCl was observed in the 35 h duration test (Fig. S33†), further revealing the stable durability. By using Pt species as the co-catalyst, TpPaCl showed a high AQE of 7.54% at 420 nm (Fig. S34 and S35†). The HER rate and AQY remained at an advanced level when compared to various state-of-the-art photocatalysts (Fig. 2h, Tables S9 and S10†). The AQYs recorded at 450, 500, 550, and 600 nm were 6.1%, 1.96%, 1.03%, and 0.84%, respectively, indicating the consistency with light adsorption band. In addition, TpPaCl exhibited a higher HER rate in seawater than in pure water in the presence of a Pt co-catalyst (Fig. 2f). Moreover, through the optimization of the catalyst dosage (Fig. S36 and S37†), TpPaCl exhibited an HER rate of up to 188.1 mmol g^−1^ h^−1^ in seawater in the presence of the Pt co-catalyst, which is higher than that in pure water (137.6 mmol g^−1^ h^−1^).

### HER thermodynamics

**Fig. 3:**
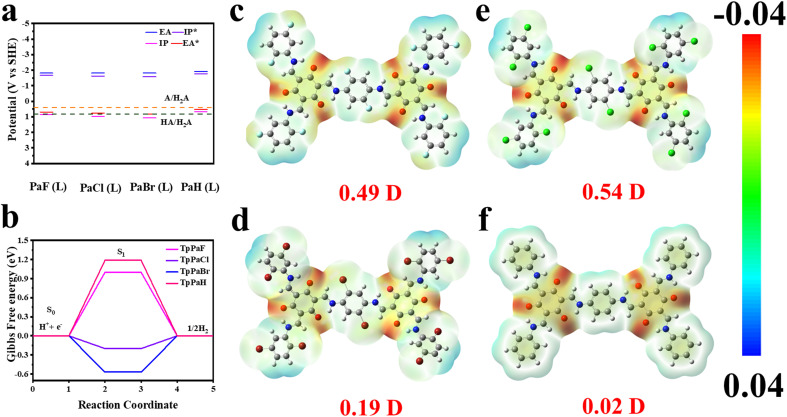
(a) (TD-)DFT calculation predicted ionization potential (IP), electron affinity (EA), and excited states (IP* and EA*) on the cut-out cluster models in water. PaF(L), PaCl(L), PaBr(L), and PaH(L) are representative fragments of TpPaF, TpPaCl, TpPaBr and TpPaH, respectively. (b) Hydrogen-bonding free energy calculated at halogen substituted carbon catalytic sites of TpPaX. The electrostatic potential distribution of (c) TpPaF, (d) TpPaCl, (e) TpPaBr, and (f) TpPaH.

The thermodynamic nature of TpPaX primarily determines their HER behavior and was investigated by theoretical analysis and synchrotron-based soft XAS spectra. Density functional theory (DFT) and time-dependent DFT (TD-DFT) calculations using an implicit solvation model, solvation model based on density (SMD), to simulate a water–solid interface were conducted to theoretically predict the ground state and excited state properties in water plus the photocatalytic thermodynamics (Fig. S38–S41†). The corresponding representative fragments of PaF(L), PaCl(L), PaBr(L), and PaH(L) were modeled for TpPaF, TpPaCl, TpPaBr, and TpPaH, respectively. Fig. 3a illustrates the following calculated items: ionization potential (IP), electron affinity (EA), exciton ionization potential (IP*), and exciton electron affinity (EA*).^36,37^ All the EA and IP* values were more negative than the proton reduction potential, implying the favorable driving force for H_2_ evolution. The IP and EA* of TpPaCl and TpPaBr were more positive than the one-hole (HA/H_2_A) and two-hole (A/H_2_A) oxidation potentials of ascorbic acid, corresponding to their superior HER activity. By contrast, TpPaF has an IP positive than the A/H_2_A potential, while the EA* is located between HA/H_2_A and A/H_2_A potentials. The IP and EA* of TpPaH(L) cannot cross the A/H_2_A potential, and the IP of TpPaH(L) is far away from the A/H_2_A potential. As a result, the low photocatalytic HER performance was observed for TpPaF and TpPaH, due to thermodynamic limitations.

Hydrogen-bonding energies of H atom adsorption on the possible active sites of TpPaX were calculated by using the corresponding fragments (Fig. S42–S45†). In a general photocatalytic HER process, under an acidic environment, H_2_ is generated through three steps involving the successive formation of (1) the initial state H^+^ + e^−^, (2) the adsorption state of H* on the catalyst surface, and (3) the final state of 1/2H_2_ for the desorption.^38,39^ The closer Gibbs free energy of H* adsorption (Δ*G*_H*_) to zero is preferred in the HER process, while the more positive and negative Δ*G*_H*_ will, respectively, hamper the H adsorption and H_2_ desorption. Surveying the H adsorption sites indicated that the most favourable active sites are the C atoms adjacent to the halogen atoms in the repeating units of the TpPaCl and TpPaBr frameworks, and the O atoms in the TpPaF and TpPaH frameworks. The minimum Δ*G*_H*_ values for H adsorption on the carbon/oxygen (C/O) atoms of TpPaX are summarized in Fig. 3b. The Δ*G*_H*_ for the most possible H adsorption sites on TpPaH and TpPaF are, respectively, identified to be 1.19 and 1.0 eV, reflecting the weak H* adsorption. By contrast, the H* adsorption on the carbon atom adjacent to the halogen atom of TpPaCl and TpPBr exhibited the negative Δ*G*_H*_ with the values of −0.2 and −0.57 eV, respectively. The closest Δ*G*_H*_ of TpPaCl to zero facilitates both the H* adsorption and H_2_ release, allowing the feasible HER in the absence of the Pt co-catalyst. The electron distribution images of TpPaX showed that the largest dipole moment of 0.54 debye is for TpPaCl (Fig. 3c–f). The larger dipole moment reflects the stronger polarization, benefiting the charge separation and creation of favorable electron transfer channels for the stabilization of the photogenerated electrons and timely transport to the active sites for successive interaction with the adsorbed protons.

**Fig. 4:**
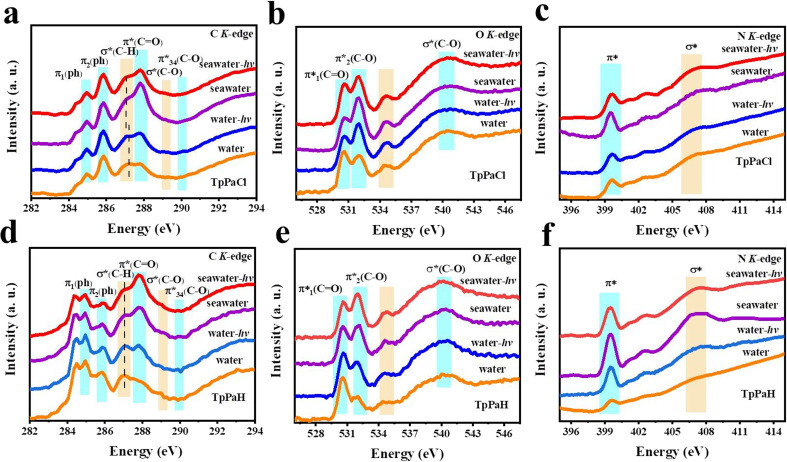
(a and d) C, (b and e) O, and (c and f) N K-edge XANES spectra for TpPaCl and TpPaH. Water, water-*hv*, seawater, and seawater-*hv*, respectively, denote that the sample was dispersed in pure water, pure water under visible-light illumination, seawater, and seawater under visible-light illumination. After that, the solid was collected by filtration and dried for the following measurements.

Soft XAS spectra were collected to gain insight into the active sites by monitoring the structural variation of TpPaCl and TpPaH under various conditions (Fig. 4): (1) water, the sample was dispersed in pure water; (2) water-*hv*, the sample was dispersed in pure water under visible-light illumination; (3) seawater, the sample was dispersed in seawater; (4) seawater-*hv*, the sample was dispersed in seawater under visible-light illumination. After the respective pre-treatment, the solid was isolated by filtration and dried for the collection of XANES spectra, as displayed in Fig. 4a. C K-edge XANES spectra of TpPaCl (water) showed three peaks at 284.9, 285.8, and 290.0 eV, attributable to the excitations of the C=C π-antibonding orbital of the benzene ring (π*(Ph)).^39–41^ The peaks at 287.2, 287.8, and 289.3 eV, respectively, corresponded to the excitations of the C–H/Cl antibonding orbital (σ*(C–H/Cl)), the C=O π antibonding orbital (π*(C=O)), and the C–O antibonding orbital (σ*(C–O)).^40,41^ After illumination, the σ*(C–H/Cl) bond of TpPaCl (water-*hv*) shifted to a lower binding energy relative to TpPaCl (water), corresponding to the gathering of the photogenerated electrons on the carbon atoms of the benzene ring.^42^ A similar phenomenon was observed by comparing the C K-edge XANES spectra of TpPaCl (seawater) and TpPaCl (seawater-*hv*). Notably, the negative shifting of the σ*(C–H/Cl) bond was also found from TpPaCl (water) to TpPaCl (seawater), suggesting the increase of the electron density in the carbon atom that will promote the H* adsorption. This alternation comes from the adsorption of certain salts in seawater. For example, the XPS spectrum of TpPaCl after adsorbing NaCl showed the residue of NaCl (Fig. S46†). Compared with TpPaCl, TpPaH demonstrated the σ*(C–H) peak with slightly lower binding energy, corresponding to the influence of Cl inclusion on the electronic state of the carbon atom. Nonetheless, negligible shifting of the σ*(C–H) peak and other signals was found over TpPaH after adsorbing salts and illumination (Fig. 4d), in line with the DFT calculation that these carbon atoms are not the potential active sites in the HER. The O K-edge XANES spectrum of TpPaCl showed three peaks at 530.7 eV and 532.8 eV, assignable to the excitations of π*(C=O), and 540.5 eV from the C–O antibonding orbital (σ*(C–O)) (Fig. 4b).^41^ The N K-edge XANES spectrum of TpPaCl illustrated the peaks at 399.6 eV and 407.2 eV, respectively, for excitations of π*(C–N) and σ*(C–N) (Fig. 4c).^43^ After adsorption of salts or illumination, only the intensity of π*_2_(C–O) and π*(C–N) peaks emerged in the O and N K-edge XANES spectra of TpPaCl, meaning the enhanced orbital hybridization while excluding the potential active sites (Fig. 4b and c). Similar phenomena were observed in the O and N K-edge XANES spectra of TpPaH (Fig. 4e and f). All these comparisons suggest that the carbon atoms in the benzene ring of TpPaCl serve as the HER active sites, in line with the theoretically predicted results.

### HER kinetics

**Fig. 5:**
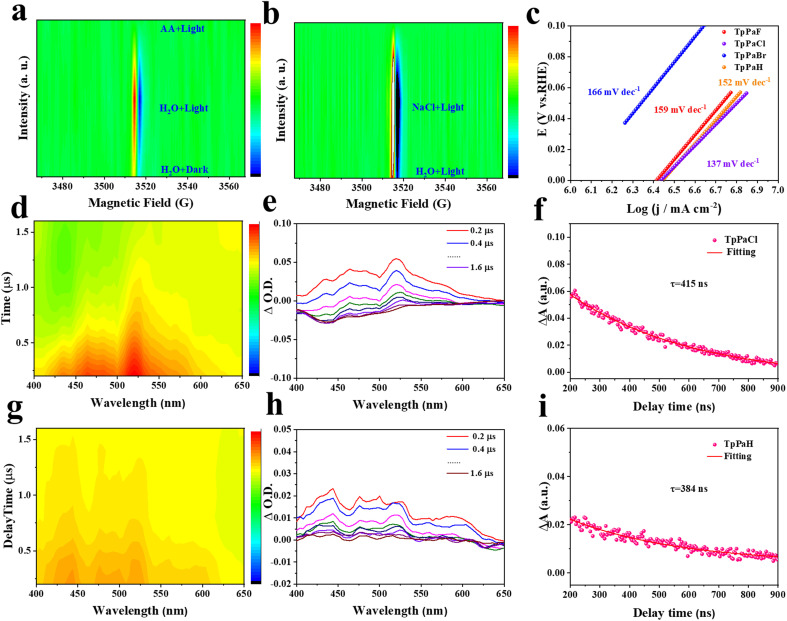
(a) 2D mapping ESR signal of TpPaCl in H_2_O in the dark and in 0.1 M aqueous AA solution under visible light illumination. (b) 2D mapping ESR signal of TpPaCl in H_2_O and 3.5 wt% aqueous NaCl solution under visible light illumination. (c) HER Tafel slopes of TpPaX. 2D mapping nanosecond transient absorption spectra of (d) TpPaCl and (g) TpPaH. Nanosecond transient absorption spectral signals of (e) TpPaCl and (h) TpPaH in 3.5 wt% aqueous NaCl solution, *λ*_ex_ = 355 nm. Decay traces of (f) TpPaCl at 520 nm and (i) TpPaH at 444 nm.

Photogenerated carrier kinetics is another important factor influencing the HER performance of TpPaX and is studied below. Quasi *in situ* ESR spectra of TpPaCl in different solutions were collected to provide more information related to the potential HER pathway. A weak ESR signal at *g* = 2.005 was observed by immersing TpPaCl in pure water in the dark, while switching to visible-light irradiation significantly strengthened this signal, attributable to the formation of more cation radicals *via* light harvesting, excitation, and separation on the TpPaCl matrix (Fig. 5a and S47a†). This ESR signal was not observed by dispersing TpPaCl in the AA solution, reflecting the rapid quenching of the cation radicals. This phenomenon suggests a free cation radical avenue for the present HER process. TpPaCl in NaCl solution displayed a stronger ESR signal than in water, suggesting the improved ability to generate cation radicals in seawater under illumination (Fig. 5b and S47b†).^44^ The decreased peak intensity was found in the PL spectrum of TpPaCl in NaCl solution relative to that in pure water (Fig. S48†). Thus, the charge recombination on TpPaCl was inhibited in seawater relative to pure water, enhancing the seawater decomposition to produce H_2_.

Linear-sweep voltammetry (LSV) curves of TpPaX recorded in aqueous Na_2_SO_4_ solution under an Ar atmosphere (Fig. S49†) demonstrated the gradually decreased overpotential in the order of X = H > F > Br > Cl, suggesting the favorable H_2_ evolution by including Cl atoms.^45^ The HER kinetics and electrochemical reduction were evaluated by Tafel plots (Fig. 5c). The smallest Tafel slope of 137 mV dec^−1^ was identified for TpPaCl, suggesting a rate-determining step (RDS) of the Volmer step.^46^ This was further reflected by the high kinetic isotope effect (KIE) value of 6.2 by comparing the HER activity using D_2_O and H_2_O (Fig. S50†). This excludes the possible RDS involving the formation of the H–H bond.^47–49^

Nanosecond transient absorption spectra of TpPaCl and TpPaH in seawater were conducted to investigate the effect of the Cl atom on the charge migration kinetics in seawater. Upon 355 nm laser excitation, TpPaCl and TpPaH demonstrated broadband absorption, respectively, at 500–550 nm and 400–450 nm, representing the excited states (Fig. 5d, e, g and h). A gradual weakening signal of the excited state was observed by prolonging the electron transition time, indicating that the catalyst in the excited state underwent a de-excitation process and returned to the ground state. The lifetimes of the charge-separated state of TpPaCl and TpPaH were obtained by fitting the decay curves (Fig. 5f and i). TpPaCl displayed a longer lifetime (415 ns) than TpPaCl (384 ns), indicating that the Cl atom in the benzene ring significantly prolonged the charge lifetime for better HER activity in seawater.^50,51^

### Influence of integrated halogen atoms

**Fig. 6:**
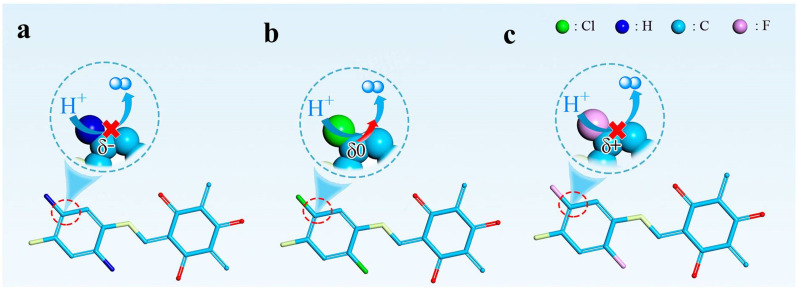
The influence of integrated halogen atoms on the HER active sites of (a) TpPaH, (b) TpPaCl, and (c) TpPaF. The Cl-lined carbon atom is close to zero valence, fascinating both the H* adsorption and H_2_ desorption.

Based on the above thermodynamic and kinetic investigations, a possible influence of the integrated halogen atoms of the constructed catalysts TpPaX on the photocatalytic HER process is proposed in Fig. 6. After light harvesting, the excited electrons were separated and transferred into the carbon atom in the benzene ring of TpPaX to capture the protons for the H* formation and successive H_2_ desorption.^52,53^ The formed cationic intermediate state oxidized the AA and returned to the original state. The architecture of TpPaH showed weak interaction with H* that inhibited the proton reduction (Fig. 6). The integration of different halogen atoms optimized both the thermodynamic and kinetic performance. From the thermodynamic viewpoint, the DFT calculations demonstrated that the halogen can finely adjust the electronic state of the nearby carbon atom in the benzene ring of TpPaX (Fig. 3b). The more the electronegativity of the halogen, the more the electron deficiency of the carbon atom near the halogen, allowing the facile modulation of the affinity towards the H atom. The Cl-linked carbon atom with a suitable electronic state allowed the feasible interaction towards protons and successive H_2_ desorption (Fig. 6b). By contrast, the too positive C atoms (Fig. 6c) or negative ones (Fig. 6a) either hinder the proton adsorption or H_2_ desorption and thus are difficult to serve as hydrogen evolution sites. From the kinetic viewpoint, the existence of halogen atoms tailors the electrostatic potential distribution of the COF and Cl atoms causing a strong polarization field that promotes the charge separation and transfer, inhibiting the charge recombination and prolonging the electron lifetime (Fig. 5c, f and i). In a seawater environment, the dipole moment of TpPaCl increased from 0.54 to 0.68, and the reduction potential of TpPaCl shifted to a more negative direction, reflecting stronger proton reduction capability while maintaining the oxidation capacity toward AA. The existence of salts in seawater led to salt adsorption which further enhanced the kinetics (Fig. 4, 5b, S49–56†). All of these enhanced the HER activity of TpPaCl in seawater.

## Conclusions

A series of halogenated and halogen-free COFs (TpPaX) were synthesized through Schiff base condensation by using the building blocks with or without halogen atoms (F, Cl, and Br). The photochemical properties plus the carrier thermodynamics and kinetics of TpPaX were finely modulated *via* this molecular engineering strategy. In particular, the integration of halogen atoms tailored the electronic state of the adjacent carbon atoms to optimize the Gibbs free energy of H* adsorption (Δ*G*_H*_), the vital thermodynamic step for the HER. Among them, the Cl atom greatly reduced the energy barrier for the formation of the H* intermediate on the adjacent carbon atoms that served as the photoactive HER sites without the assistance of the Pt co-catalyst. The carrier kinetics were also enhanced due to the strong polarization after the inclusion of electronegative halogen atoms, in which the Cl atom led to the largest dipole of the polymer skeleton to significantly improve the charge separation and transfer. Thus, the constructed Cl-tethered COF TpPaCl effectively catalyzed the visible-light-driven H_2_ production under co-catalyst free conditions. The HER rate increased to as high as 16.3/11.5 mmol g^−1^ h^−1^ by switching pure water to artificial/real seawater, attributable to the enhanced kinetics after salt adsorption. In this work, we constructed a high-performance system for photocatalytic H_2_ production and highlighted the great potential of functional COFs in designing photocatalysts at the molecular level.

## Author contributions

F. Ma and X. Chi performed experiments and wrote the manuscript. Y. Wen, Q. Yue, T. Chen, X. Yu, and X. Liu helped with the synthesis and characterization. Y. Zhou and J. Wang conceived and supervised the project. All the authors discussed the results, commented on and revised the manuscript.

## Conflicts of interest

There are no conflicts to declare.

## Supplementary Material

SC-OLF-D5SC00082C-s001

## Data Availability

The data supporting this article have been included as part of the ESI.†
