# Supplementary material for: Engineering charge transfer by tethering halogens to covalent organic frameworks for photocatalytic sacrificial hydrogen evolution
Source: Chem Sci. 2025 Jun 6;16(28):12906–16. doi: 10.1039/d5sc00082c (PMC12168954; doi:10.1039/d5sc00082c)
Supplement: SC-016-D5SC00082C-s001 [file SC-016-D5SC00082C-s001.pdf]

Supplementary Information (SI) for Chemical Science.
This journal is © The Royal Society of Chemistry 2025

Supplementary Information

For

Engineering charge transfer by tethering halogens onto covalent organic framework for photocatalytic sacrificial hydrogen evolution

Fangpei Ma,[‡a] Xiao Chi,[‡b] Ying Wen,[a] Qihong Yue,[a] Tao Chen,[a] Xiaojiang Yu,[c] Xiaoling Liu,[a] Yu Zhou[*a] and Jun Wang[*a]

[a] *State Key Laboratory of Materials-Oriented Chemical Engineering, College of Chemical Engineering, Nanjing Tech University, Nanjing, 211816, China*

[b] *Department of Physics, National University of Singapore, 117576, Singapore*

[c] *Singapore Synchrotron Light Source, National University of Singapore, 5 Research Link, 117603, Singapore*

‡ These authors contributed equally: Fangpei Ma and Xiao Chi.

Corresponding Authors

Email: njutzhouyu@njtech.edu.cn (Y. Zhou); junwang@njtech.edu.cn (J. Wang)

General experimental methods

Instrumentation

Powder X-ray diffraction (PXRD) patterns were recorded on a Smart Lab diffractometer (Rigaku Corporation) with a Cu rotating-anode source at 9 kW with the wavelengths of 1.5406 Å and 1.5444 Å. The voltage was 40 kV and the tube current were 40 mA. The scanning angle (2θ) range was from 2° to 30°, with scanning speed of 5°/min and the step size of 0.02°. The morphology of COFs was investigated using a field emission scanning electron microscope (FE-SEM; Hitachi S-4800) with the powder sample dispersed on the conductive adhesive. Transmission electron microscopy (TEM) images were obtained on a JEOL (JEM-2100F, 200 kV) field-emission transmission electron microscope. The point resolution was ⩽ 0.23 nm and the line resolution was ⩽ 0.14 nm. The powder sample was dispersed in ethanol and then dropped on 300-mesh carbon-coated copper grid. A BELSORP-Max analyzer was exploited in the collection of nitrogen (N_2) sorption isotherms at 77 K for the analysis of surface areas and porosity. The Brunauer-Emmett-Teller (BET) method was employed to analyze the nitrogen desorption data to obtain the average pore diameter of the sample by using the equation of $D=4V_P/S$ (S: specific surface area, V_P: pore volume, and D: pore diameter). The pore size distribution profiles were calculated by using the non-local density functional theory (NLDFT) with the cylindrical pore model. The pore size dispersion curve revealed the position of the most probable pore size. X-ray photoelectron spectroscopy (XPS) spectra were recorded on an X-ray photoelectron spectrometer (AXIS Supra) outfit with Al Kα radiation. The binding energy was calibrated through C1s at 284.8 eV. The scanning range was from 0 eV to 1500 eV and the step size was 0.2 eV. Fourier transform infrared spectra (FTIR) of raw materials and COFs were collected with an Agilent Cary 660 instrument by using a Deuterated Triglycine Sulfate (DTGS) detector. The scanning range of the mid-infrared region is 4000-400 cm^{-1}. The resolution of the FTIR instrument was 1-4 cm^{-1}, and the number of scans was 64 times. The aperture size was 1 cm^2. During the measurement, 1 mg of the sample was thoroughly ground and mixed evenly with 150 mg of dry potassium bromide (KBr) powder in an agate mortar. Then, the mixture was placed in a tablet press and pressed into a transparent thin sheet under a 10 MPa pressure for the following test. UV-vis spectra were collected with a Shimadzu UV 2600 spectrometer with $BaSO_4$ as the internal standard. External single detector was adopted, with a slit width of 5.0 nm. An RF-5301/PC

spectrofluorophotometer (Shimadzu, Japan) was involved in the collection of photoluminescence (PL) emission spectra at room temperature. Time-resolved PL decay spectra were recorded in an FLS1000 fluorescence lifetime spectrophotometer (Edinburgh Instruments, UK). Elemental analyses were carried out on a Vario EL cube CHN elemental analyzer. The combustion temperature was 1000 °C. The carrier gas was helium with a flow rate of 200 mL/min. Thermogravimetric analysis (TGA) was collected with an STA409 instrument using a heating rate of 10 °C min^{-1} to 800 °C under a dry N_2 atmosphere. Solid-state ^{13}C cross-polarization (CP)/magic-angle-spinning (MAS) nuclear magnetic resonance (NMR) spectra were recorded on an AVABCE-III NMR spectrometer (Bruker, Switzerland) with a spinning rate of 6 kHz, a contact time of 2.0 ms, and a recycle delay of 15.0 s. The solid sample was grinded into fine powder and dried under vacuum to remove the water. After that, the sample was carefully filled into a ceramic NMR rotor to be collect the NMR spectra. Water vapor isotherms were determined by a BELSORP-Max analyzer at 298 K. The content of possible residue noble metals (Pd, Pt, and Au) was measured by inductively coupled plasma-mass spectrometry (Agilent 7850).

Photoelectrochemical measurements

Photoelectrochemical properties were conducted by using a CHI 760E electrochemical workstation (Shanghai Chen-hua, China) with a Pt foil as the counter electrode, Ag/AgCl (saturated KCl) as the reference electrode, the FTO glass coated catalyst as the photoelectrode, 0.5 M aqueous Na_2SO_4 solution as the electrolyte. The slurry was prepared by mixing 5 mg of COF with 440 μL of 10% Nafion ethanol solution. The working electrode was prepared by drop-casting 88 μL of the above slurry on carbon paper (1×1 cm^2) to cover a 1 cm^2 area and dried completely. For photocurrent measurement, a 300 W Xe lamp (Perfect Light PLSSXE 300) equipped with a 420 nm cut off filter ($\lambda > 420$ nm) was used as the illumination source. Electrochemical impedance spectroscopy (EIS) was measured at open-circuit voltage with an Alternating Current (AC) amplitude of 5 mV in the frequency range of 0.1 Hz to 100 kHz.

***Quasi in-situ* electron paramagnetic resonance**

Quasi in-situ electron paramagnetic resonance (ESR) experiments were performed on Bruker EMXmicro spectrometer. Before the ESR measurement, a suspension containing the COF powder

and the solvent (H_2O/0.1 M AA solution/3.5 wt% NaCl solution, 1 mg/mL) was added to the quartz tube, and the test temperature was set at 298 K. The ESR signals were collected on a Bruker EMXmicro spectrometer under illumination by using a 300 W Xe lamp as the light source ($\lambda > 420$ nm). For comparison, the signal was also collected in the dark.

Transient absorption spectroscopy (TAS)

Transient absorption spectroscopy (TAS) spectra were measured with a Laser Flash photolysis (LFP, Dalian Institute of Chemical Physics) under room temperature. The sample (5 mg) and NaCl solution (5 mL, 3.5 wt%) were mixed and ultra-sonicated for 30 min to give a relatively homogeneous slurry. The slurry (1 mg/mL) was excited at 355 nm pulses from Nd:YAG nanosecond laser system (Nimma900, Beamtech Optronics Co., Ltd.). A pulsed 450W xenon lamp (PL450, Dalian Institute of Chemical Physics) was used as the probe light. The single-wavelength transient absorption kinetics was detected with a photomultiplier tube (R928, Hamamatsu), and the transient curve was recorded by a digital oscilloscope (Tektronix, MDO3052).

***Soft* X-ray absorption spectroscopy experiments**

Soft X-ray absorption spectroscopy (XAS) analysis was performed on the Surface, Interface, and Nanostructure Science (SINS) beamline of the Singapore Synchrotron Light Source, equipped with a Scienta R4000 electron energy analyzer.

Computational Methods

All calculations were generated with the Gaussian 09 program package. All investigated structural models were optimized with B3LYP/def2-SVP level of theory[1,2]. Grimm's empirical D3 dispersion correction with Becke-Johnson damping (D3BJ) was used in these calculations. The effect of water solvation was simulated by using the PCM/SMD solvation model[3]. We evaluated the redox potential of the different molecular fragments representing the COFs relative to H^+/H_2 redox potential. The calculation of the ionization potential (IP) and electron affinity (EA) was performed with B3LYP/def2-SVP level of theory. Time-dependent DFT (TD-DFT) was used to evaluate the exciton potentials (i.e., exciton ionization potential (IP*) and exciton electron affinity (EA*)). Charge distribution over optimized structural models was calculated by B3LYP/Def2-SVP level of theory.

Gibbs free energy change for H* adsorption (ΔG_{H*}) was calculated *via* the following equation:

$$\Delta G_{H^*} = \Delta E + ZPE - T\Delta S$$

Where *ZPE* is the zero-point energy difference and ΔS is the entropy difference between hydrogen adsorption and hydrogen in the gas phase.

Owing to the effects of the catalyst on both *ZPE* and **Δ***S* being negligible, their values can be directly obtained from references without considering the contribution of the catalyst. Then, the Gibbs free energy change for H* adsorption can be calculated *via* the following equation:

$$\Delta G_{H^*} = \Delta E + 0.24$$

$$\Delta E = E_{H-cOF} - E_{COF} - \frac{1}{2}E_{H_2}$$

Where $E_{H\text{-}COF}$ is the energy of a hydrogen atom adsorbed on the COF surface and E_{COF} is the energy of COF, and E_{H2} is the energy of a hydrogen molecule.

Results and Discussion

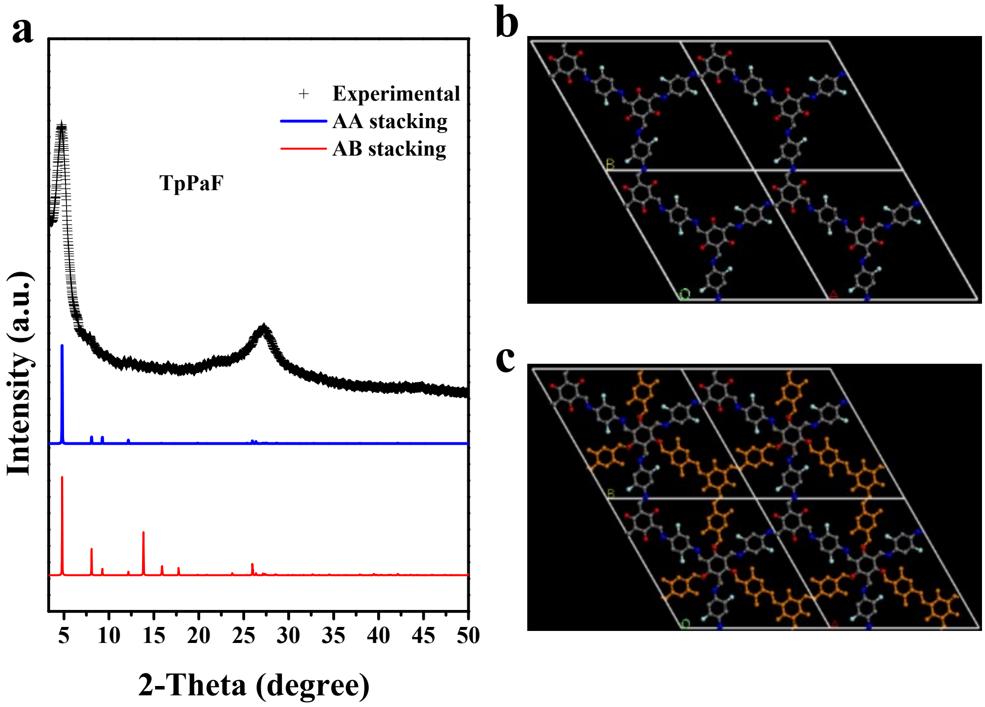

Fig. S1. (a) Experimentally observed PXRD patterns (black) of TpPaF and the simulated ones of AA-stacking (blue), AB-stacking (red). (b) Top view of simulated AA stacking mode and (c) AB stacking mode for TpPaF crystal structure.

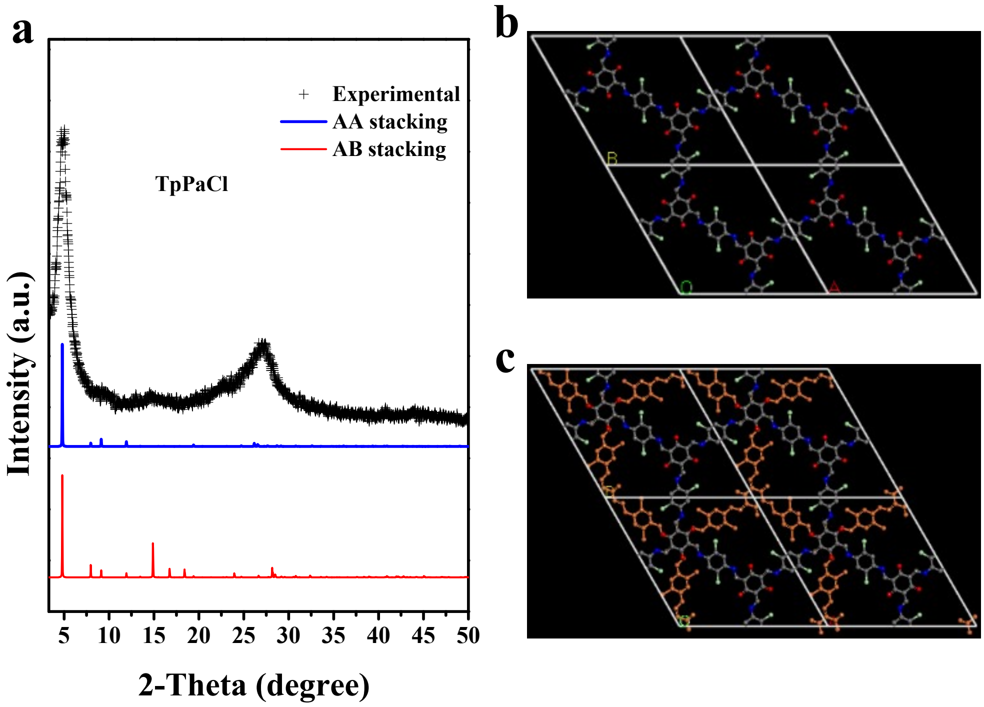

Fig. S2. (a) Experimentally observed PXRD patterns (black) of TpPaCl and the simulated ones of AA-stacking (blue), AB-stacking (red). (b) Top view of simulated AA stacking mode and (c) AB stacking mode for TpPaCl crystal structure.

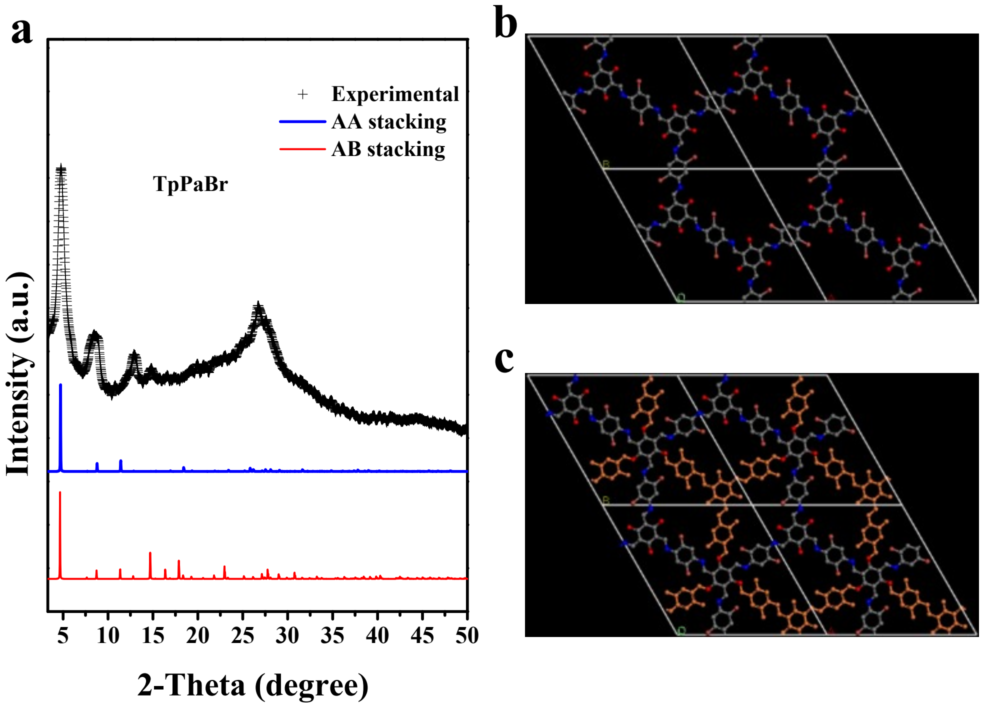

Fig. S3. (a) Experimentally observed PXRD patterns (black) of TpPaBr and the simulated ones of AA-stacking (blue), AB-stacking (red). (b) Top view of simulated AA stacking mode and (c) AB stacking mode for TpPaBr crystal structure.

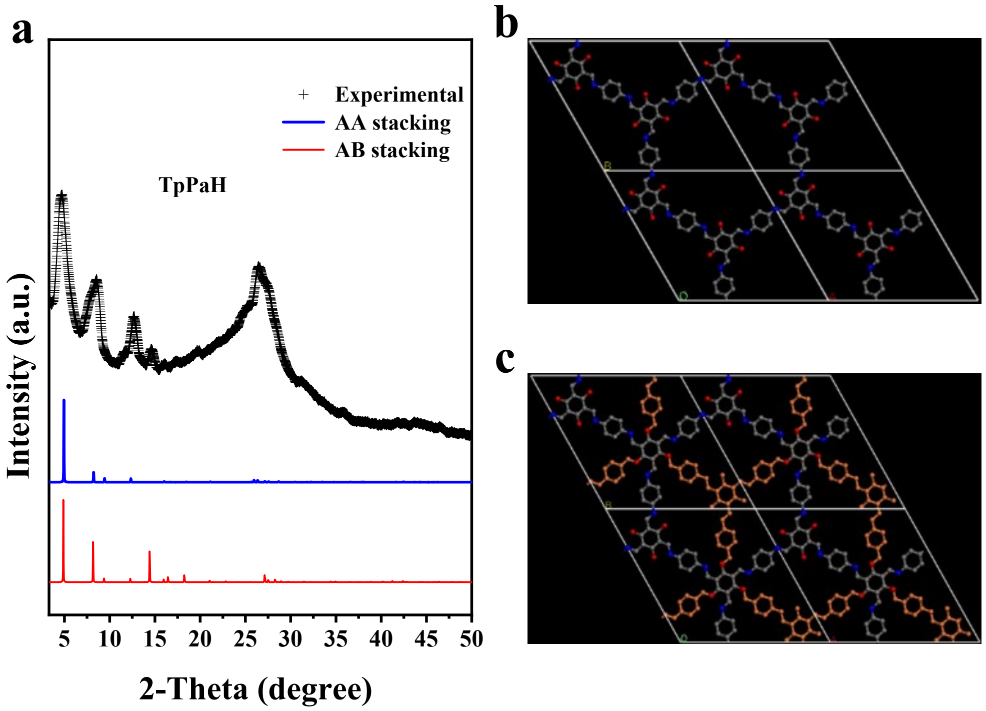

Fig. S4. (a) Experimentally observed PXRD patterns (black) of TpPaH and the simulated ones of AA-stacking (blue), AB-stacking (red). (b) Top view of simulated AA stacking mode and (c) AB stacking mode for TpPaH crystal structure.

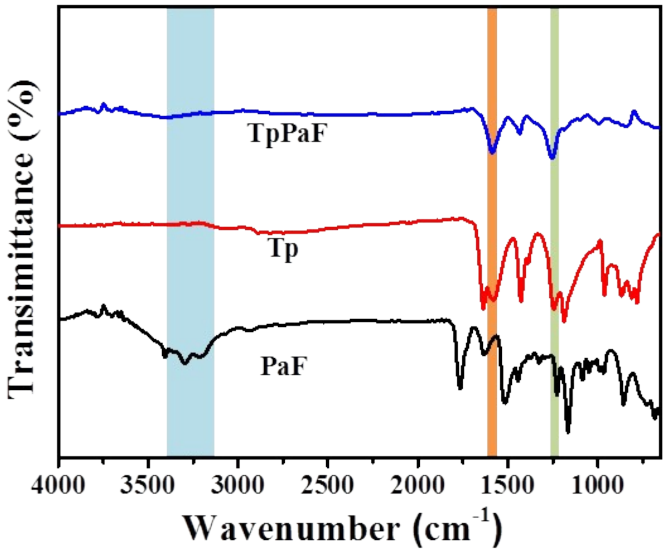

Fig. S5. FT-IR spectra of Tp, PaF, and TpPaF.

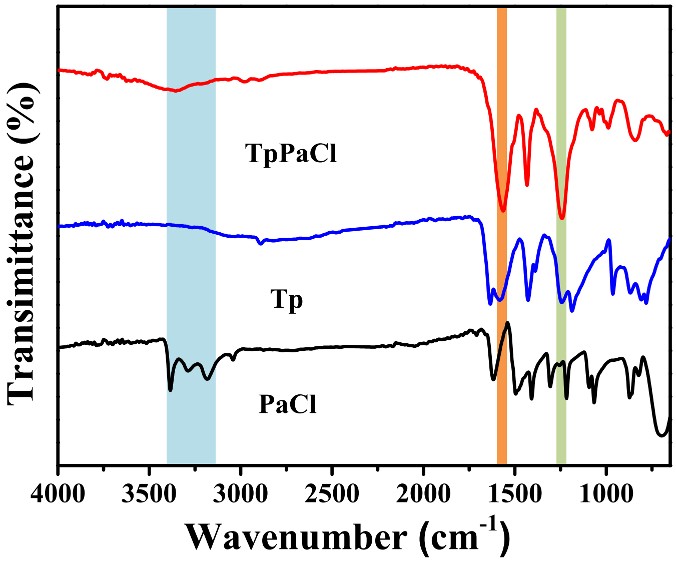

Fig. S6. FT-IR spectra of Tp, PaCl, and TpPaCl.

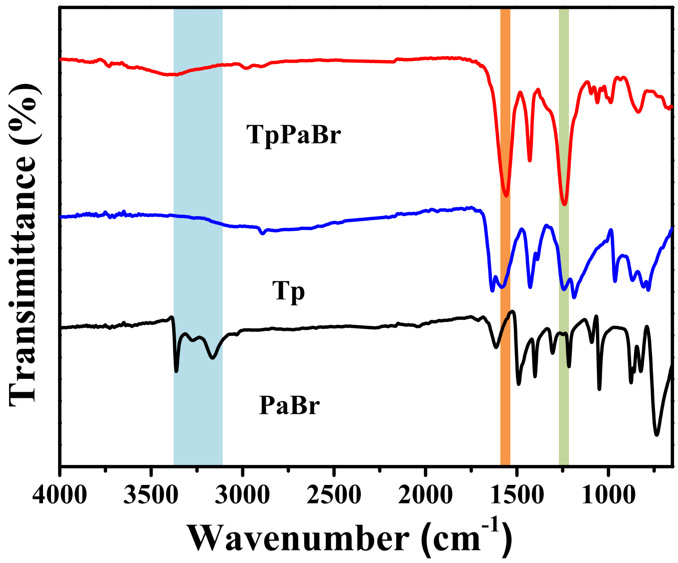

Fig. S7. FT-IR spectra of Tp, PaBr, and TpPaBr.

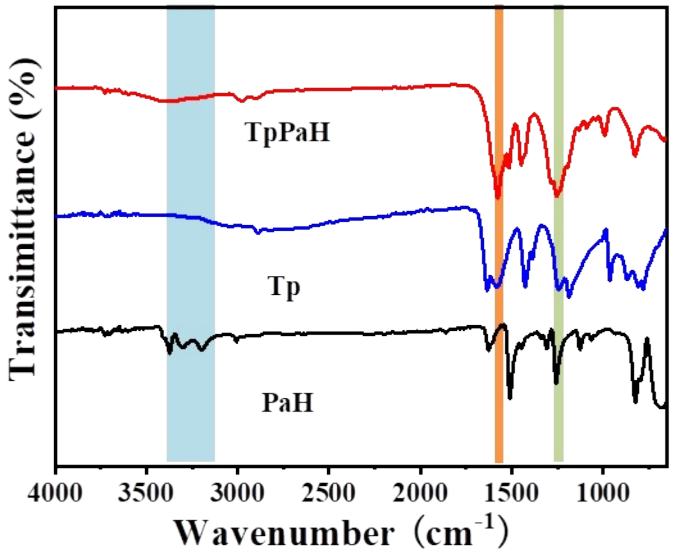

Fig. S8. FT-IR spectra of Tp, PaH, and TpPaH.

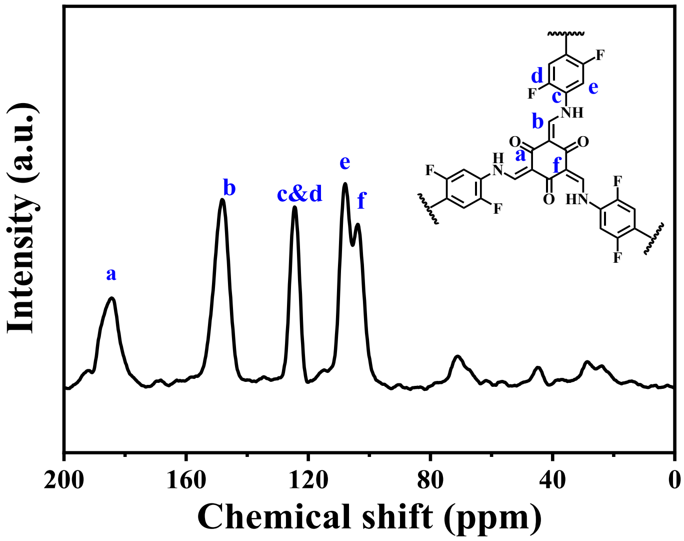

Fig. S9. Solid-state ^{13}C CP/MAS NMR spectrum of TpPaF.

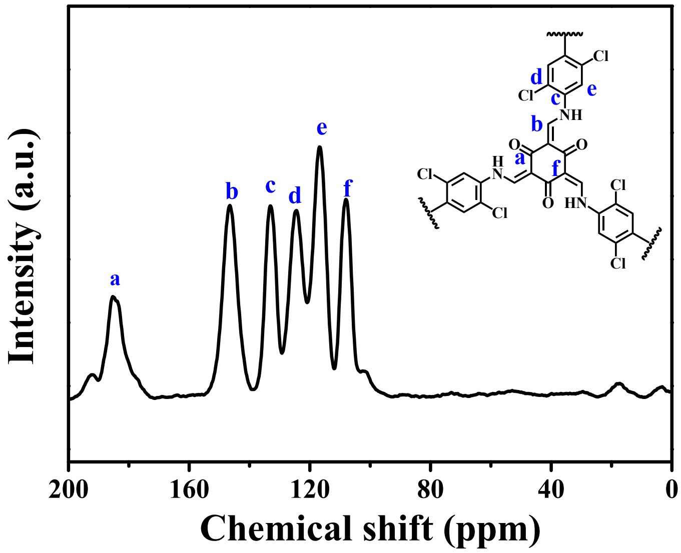

Fig. S10. Solid-state ^{13}C CP/MAS NMR spectrum of TpPaCl.

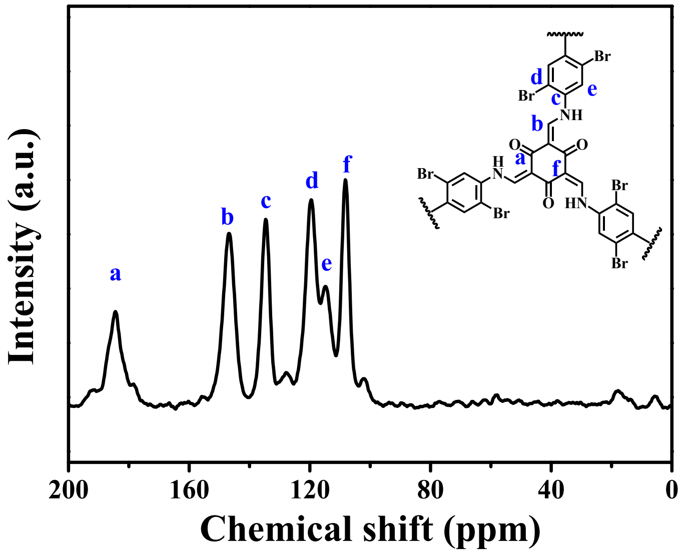

Fig. S11. Solid-state ^{13}C CP/MAS NMR spectrum of TpPaBr.

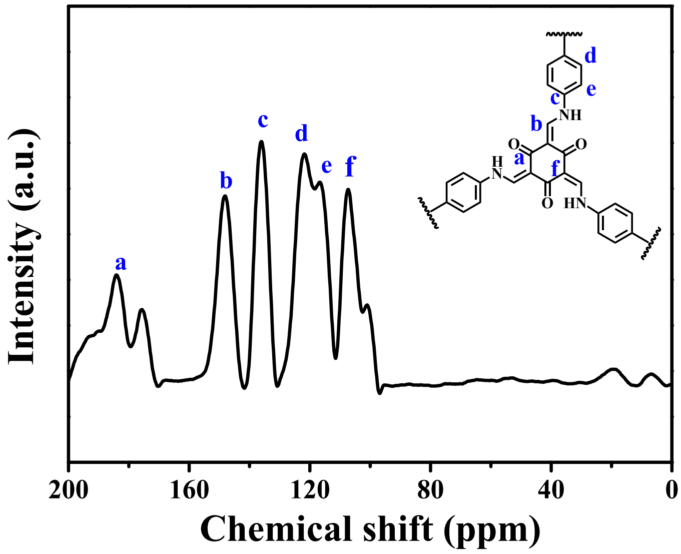

Fig. S12. Solid-state ^{13}C CP/MAS NMR spectrum of TpPaH.

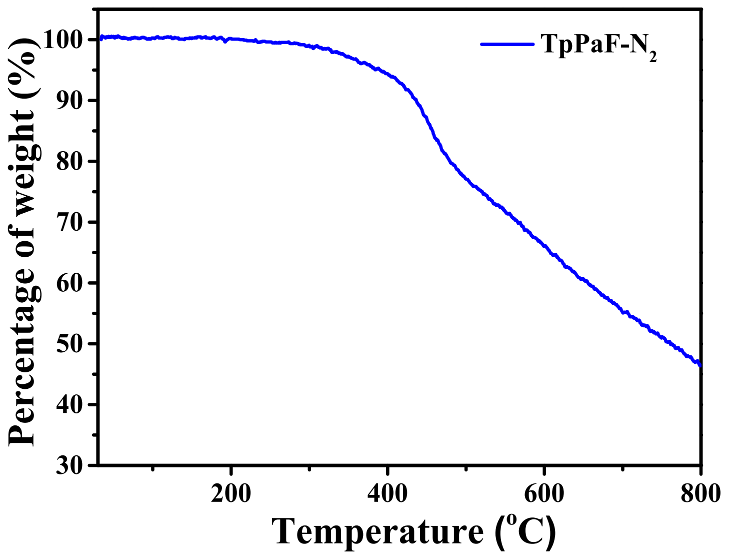

Fig. S13. TGA profile of TpPaF under N_2 atmosphere.

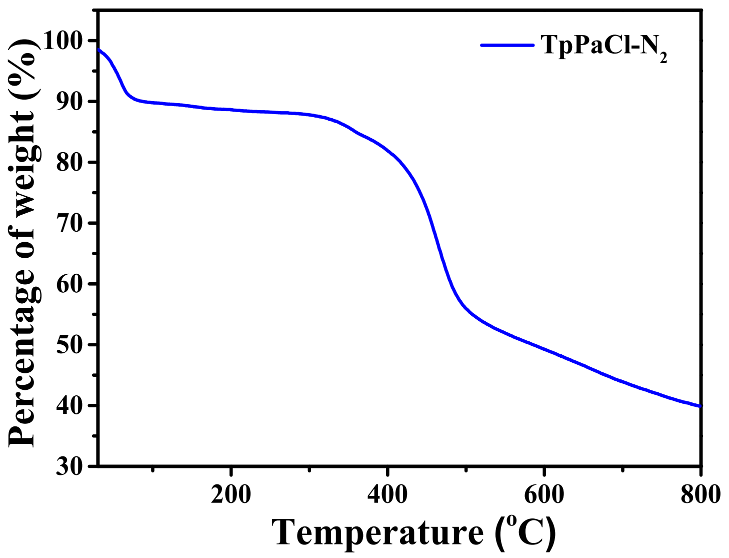

Fig. S14. TGA profile of TpPaCl under N_2 atmosphere.

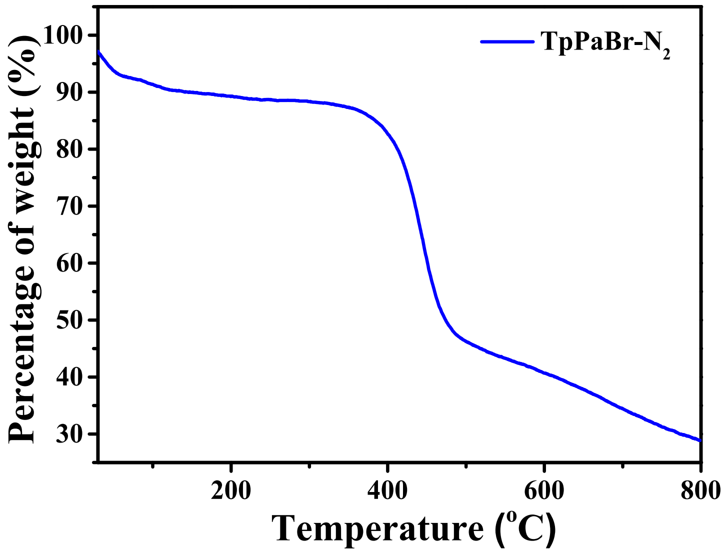

Fig. S15. TGA profile of TpPaBr under N_2 atmosphere.

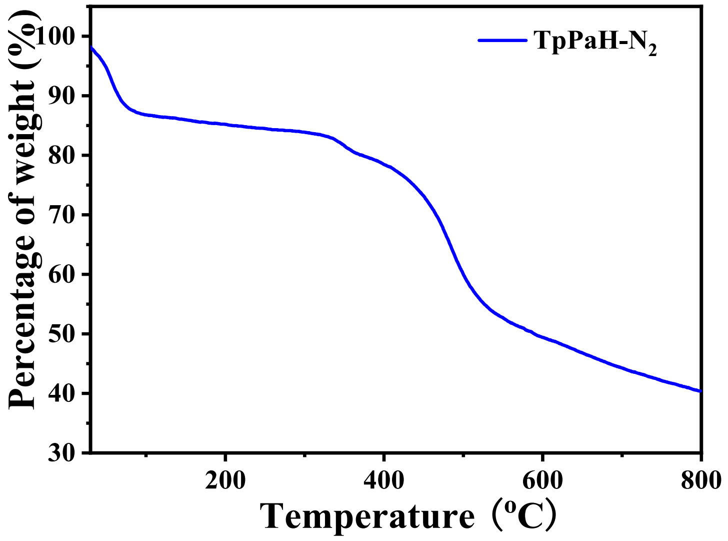

Fig. S16. TGA profile of TpPaH under N_2 atmosphere.

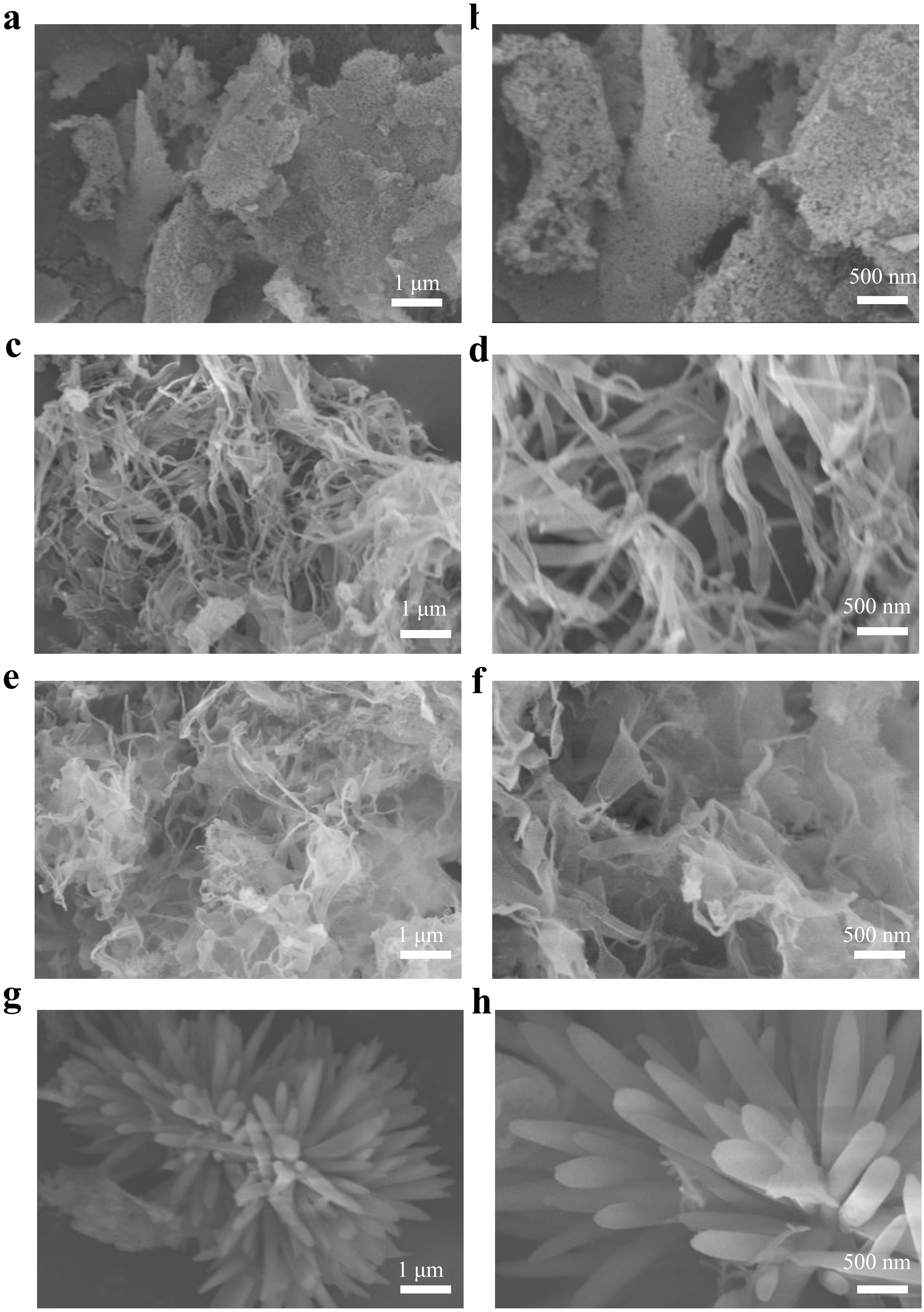

Fig. S17. SEM images of (a, b) TpPaF, (c, d) TpPaCl, (e, f) TpPaBr, and (g, h) TpPaH.

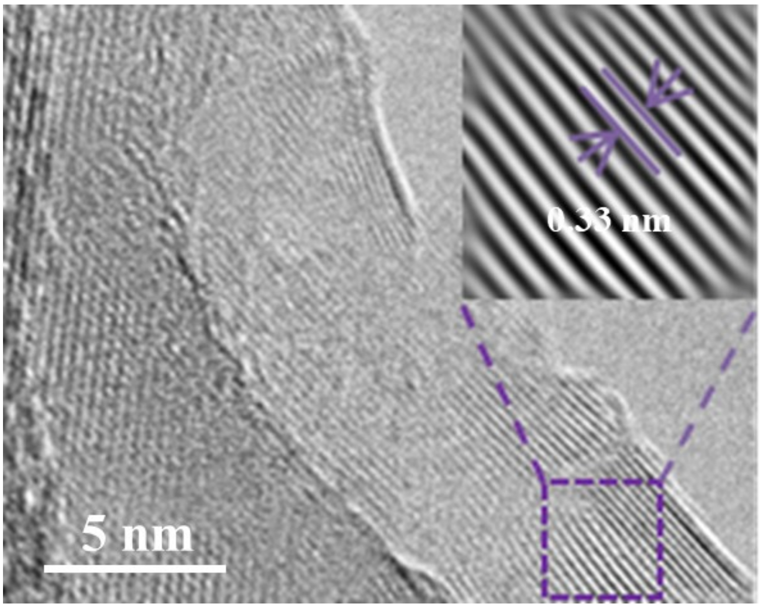

Fig. S18. HR-TEM and Fourier transform (FFT) images of TpPaCl.

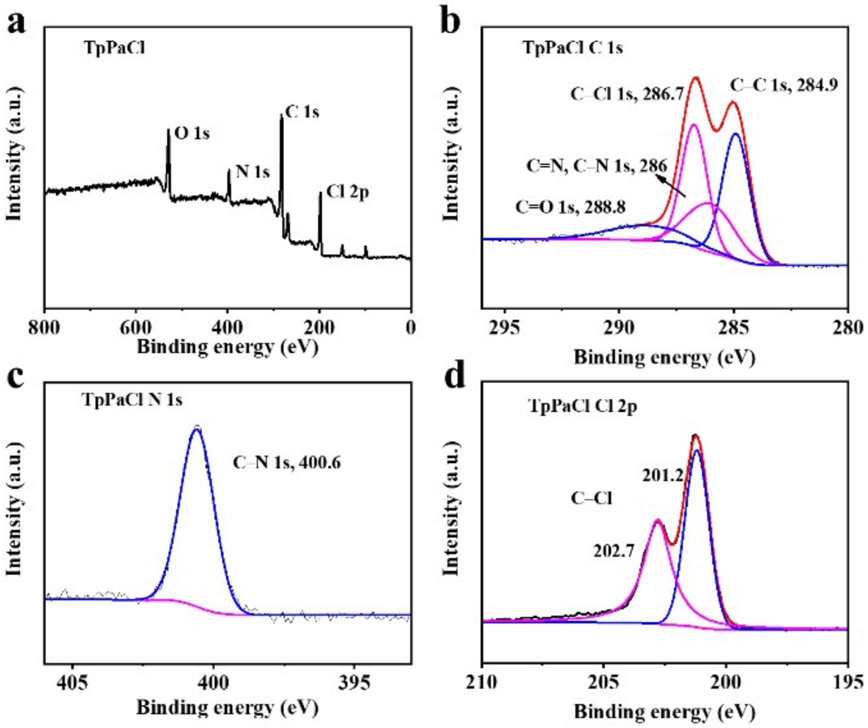

Fig. S19. (a) XPS survey scan spectra of TpPaCl. High resolution (b) C 1s, (c) N 1s, and (d) Cl 2p XPS spectra of TpPaCl.

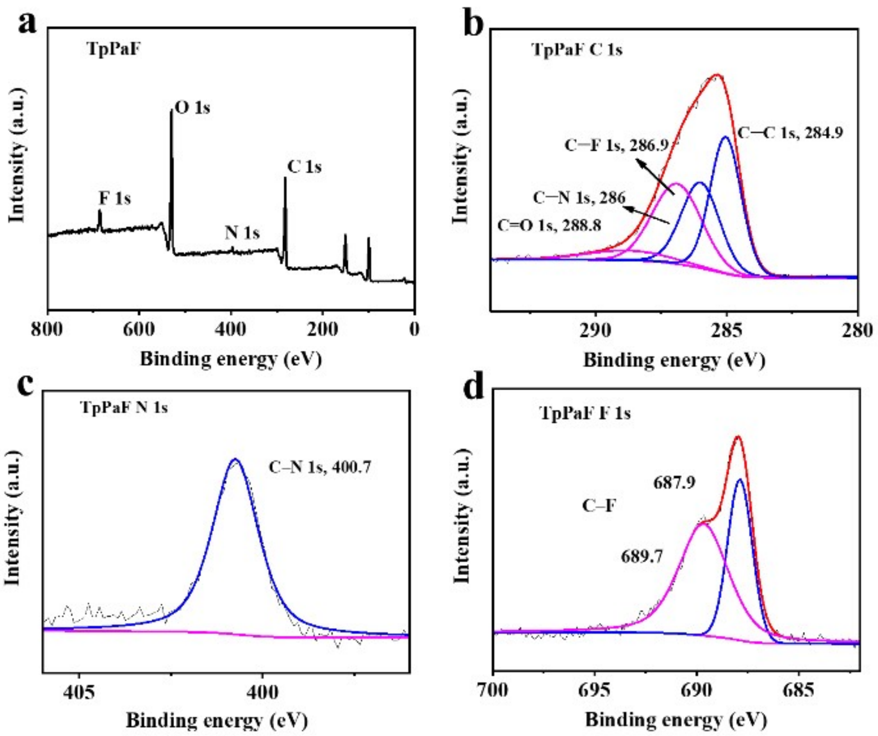

Fig. S20. (a) XPS survey scan spectra of TpPaF, high resolution (b) C 1s, (c) N 1s, and (d) F 1s XPS spectra of TpPaF.

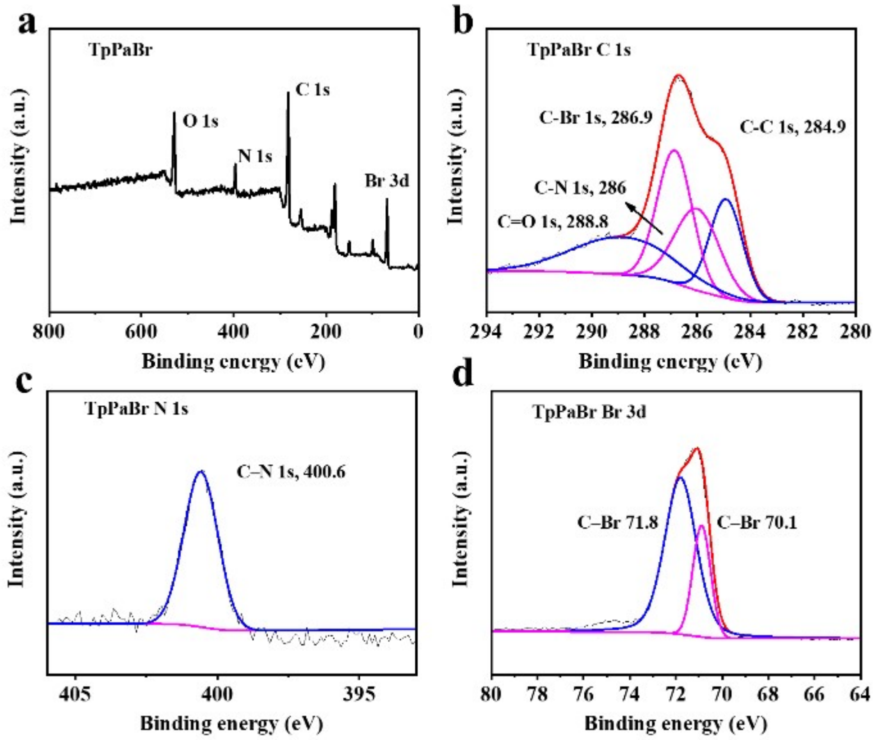

Fig. S21. (a) XPS survey scan spectra of TpPaBr. High resolution (b) C 1s, (c) N 1s, and (d) Br 3d XPS spectra of the TpPaBr.

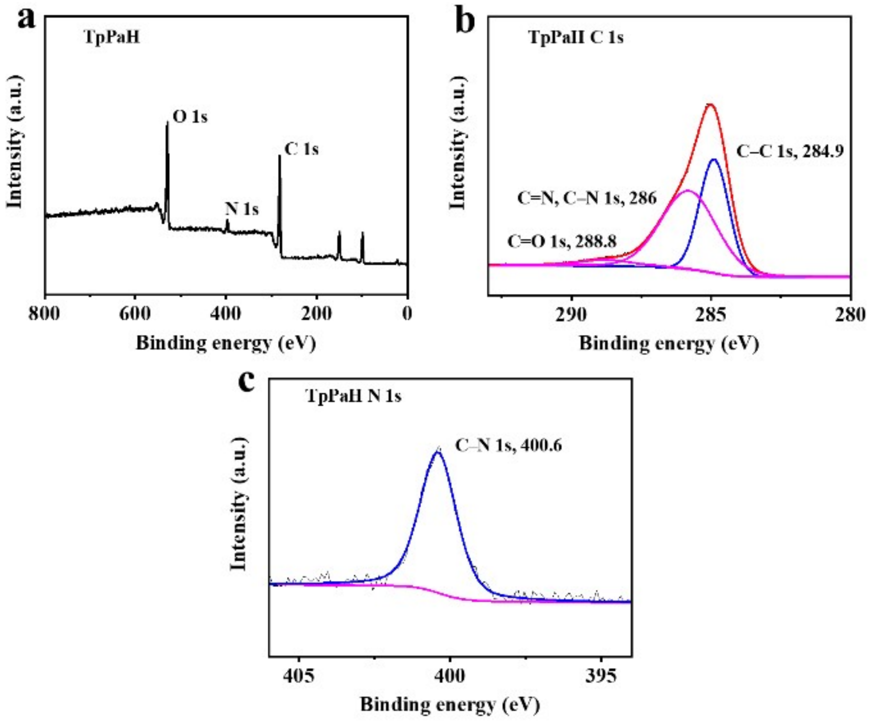

Fig. S22. (a) XPS survey scan spectra of TpPaH. High resolution (b) C 1s and (c) N 1s XPS spectra of the TpPaH.

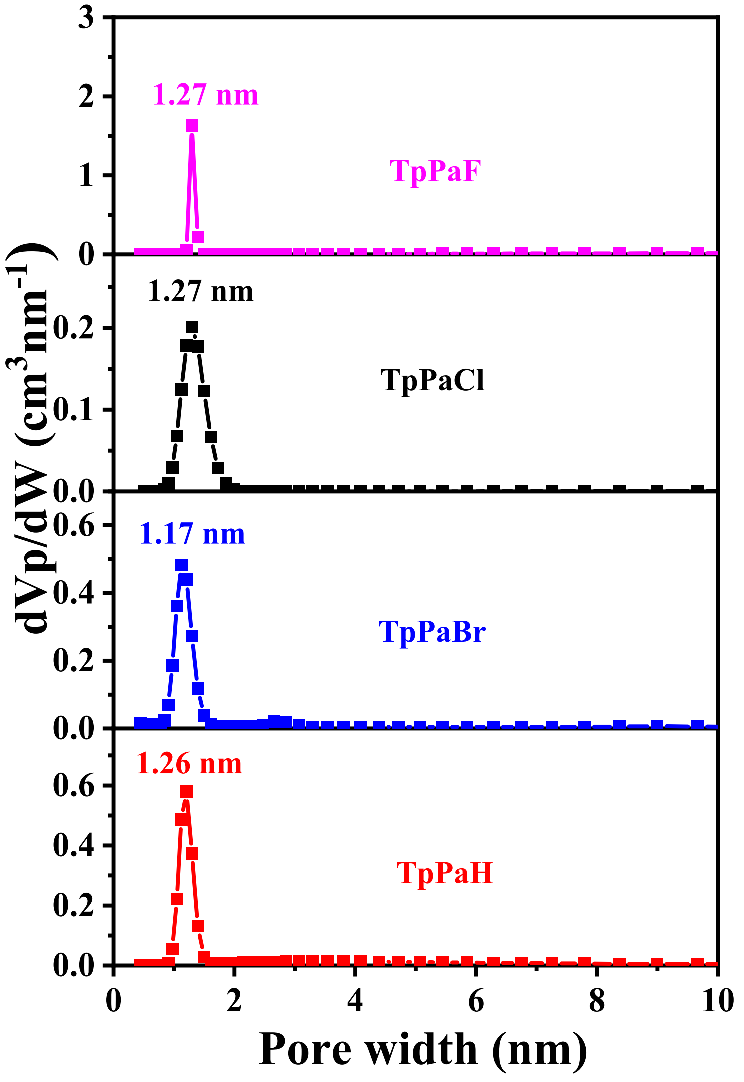

Fig. S23. The pore size distribution curves of (magenta) TpPaF, (black) TpPaCl, (blue) TpPaBr, and (red) TpPaH at 77 K.

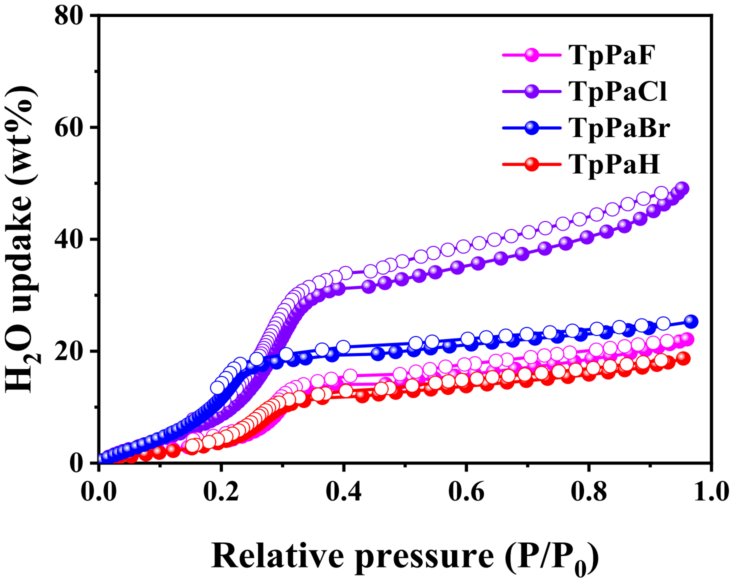

Fig. S24. Water adsorption isotherms (filled symbols) and desorption isotherms (open symbols) for TpPaF, TpPaCl, TpPaBr, and TpPaH at 298 K.

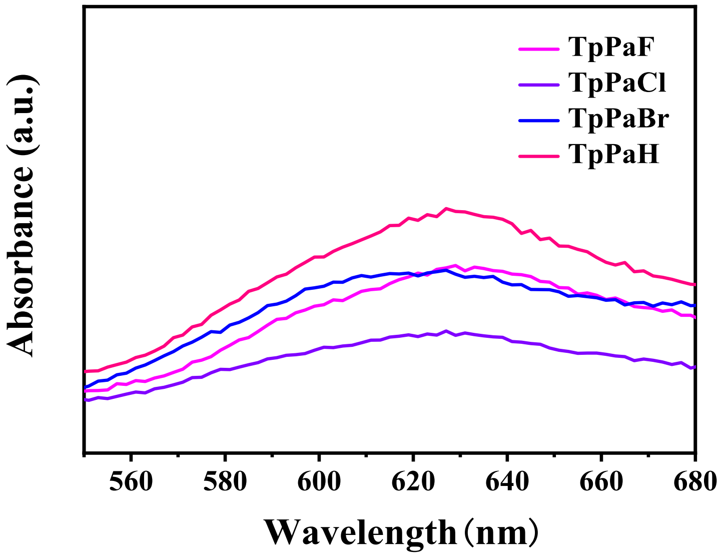

Fig. S25. Steady-state photoluminescence (PL) spectra of TpPaF, TpPaCl, TpPaBr, and TpPaH.

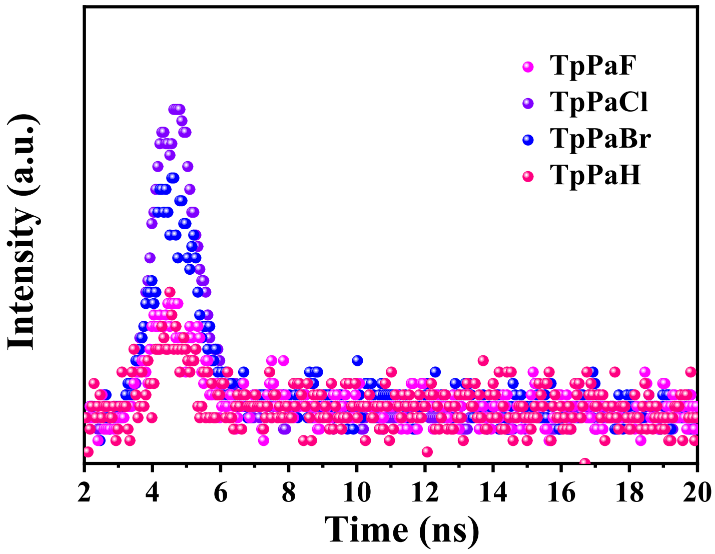

Fig. S26. Time-resolved PL decay spectra of TpPaF, TpPaCl, TpPaBr, and TpPaH.

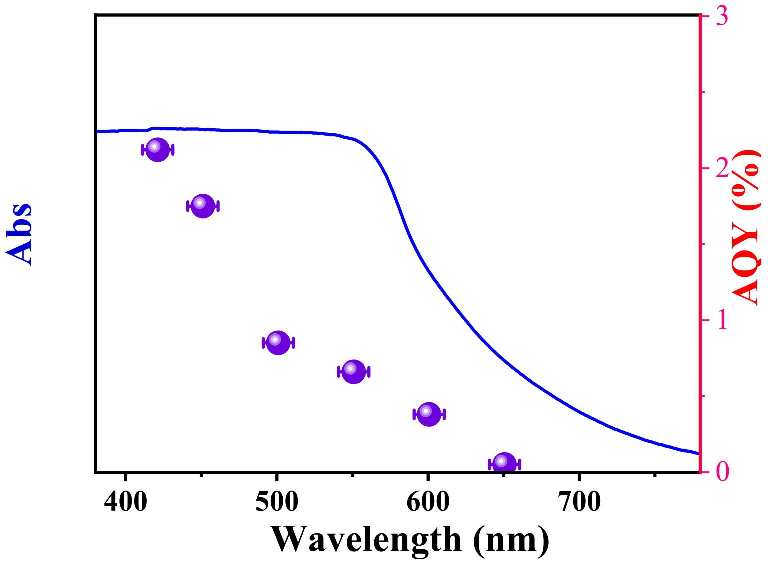

Fig. S27. Wavelength dependent AQE values of TpPaCl in seawater. An Xe-lamp equipped with a series of band-pass flitters (420, 450, 500, 550, 600, and 650 nm) was used as the light source. Reaction conditions: 5 mg TpPaCl, 0.1 M ascorbic acid, room temperature, 5 h.

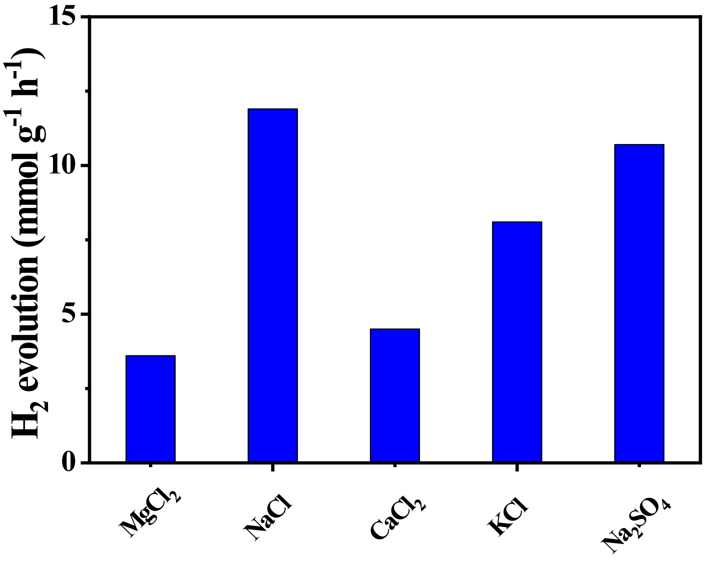

Fig. S28. Photocatalytic H_2 evolution over TpPaCl in the presence of different salts ($MgCl_2$, NaCl, $CaCl_2$, KCl, or Na_2SO_4; 3.5 wt% in the reaction solution). Reaction conditions: 2 mg TpPaCl, λ > 420 nm, 0.1 M ascorbic acid, room temperature, 5 h.

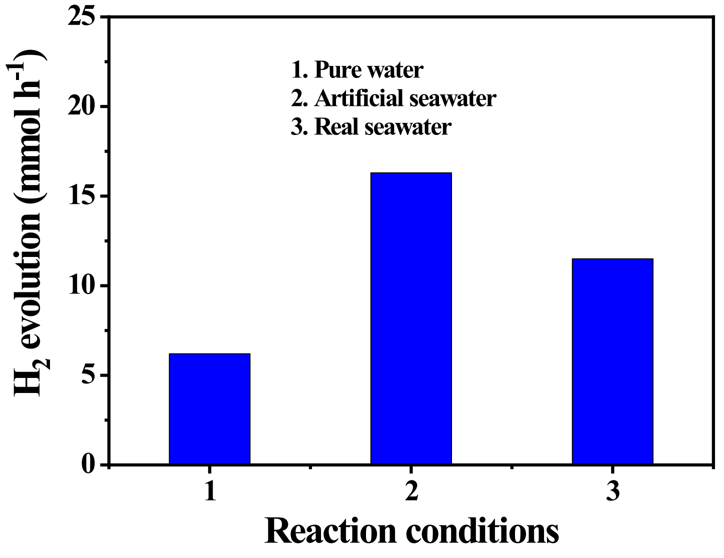

Fig. S29. Photocatalytic H_2 evolution over TpPaCl in pure water, artificial seawater, and real seawater. Reaction conditions: Reaction conditions: 2 mg COFs, λ > 420 nm, 0.1 M ascorbic acid, room temperature, 5 h. Real seawater was sampled from Qingdao, China, specifically from the waters of Lingshan Bay in Qingdao. The coordinates of the water collection point were 120.18.35.82, and the average water depth at the collection point was approximately 10 meters.

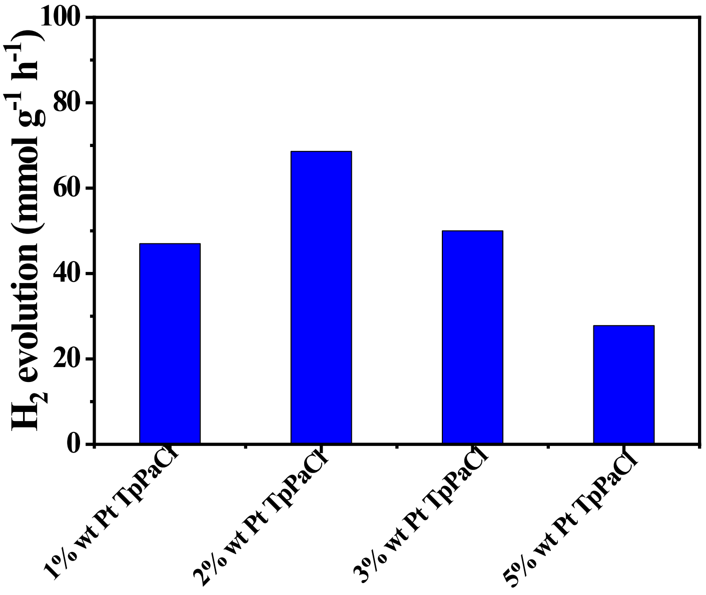

Fig. S30. Photocatalytic H_2 evolution rates of TpPaCl in pure water with different feeding amounts of hexachloroplatinic acid aqueous solution (1 wt%, 2 wt%, 3 wt%, and 5 wt% Pt relative to TpPaCl). Reaction conditions: 5 mg TpPaCl, $\lambda > 420$ nm, 0.1 M ascorbic acid, room temperature, 5 h.

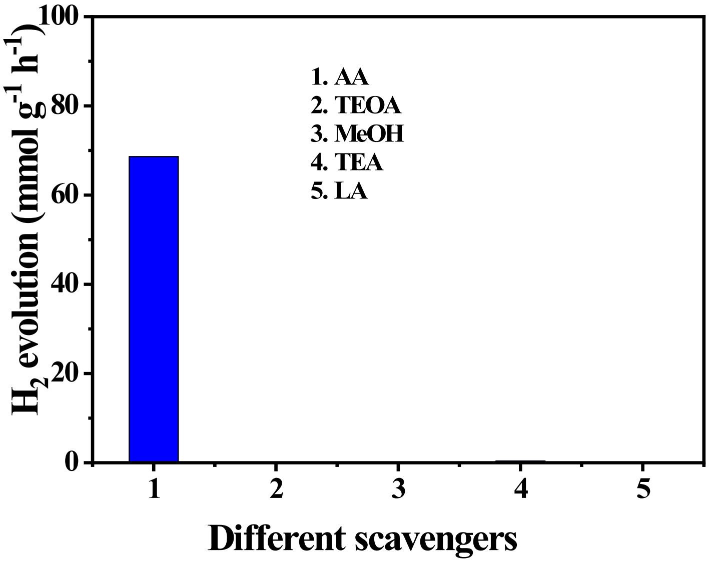

Fig. S31. Hydrogen evolution over TpPaCl in pure water by using different sacrificial agents. Reaction conditions: 5 mg TpPaCl, the aqueous solution containing sacrificial agent (1: 20 mL 0.1 M ascorbic acid; 2: 2 mL triethanolamine and 18 mL water; 3: 2 mL methanol and 18 mL water; 4: 2 mL trimethylamine solution and 18 mL water; 5: 2 mL lactic acid and 18 mL water). $\lambda > 420$ nm, hexachloroplatinic acid aqueous solution (2 wt% Pt to TpPaCl), room temperature, 5 h.

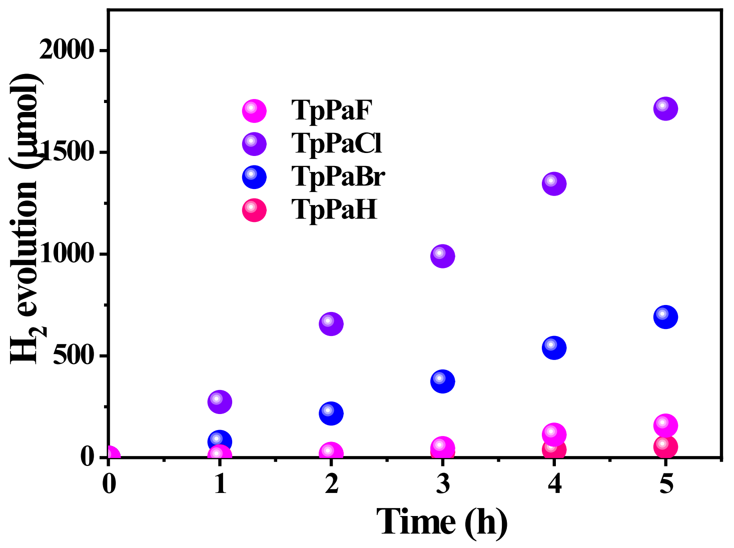

Fig. S32. Time-resolved photocatalytic H_2 evolution over TpPaF, TpPaCl, TpPaBr, and TpPaH in pure water under λ >420 nm irradiation. Reaction conditions: 5 mg TpPaX, λ > 420 nm, 0.1 M ascorbic acid, hexachloroplatinic acid aqueous solution (2 wt% Pt to photocatalyst), room temperature, 5 h.

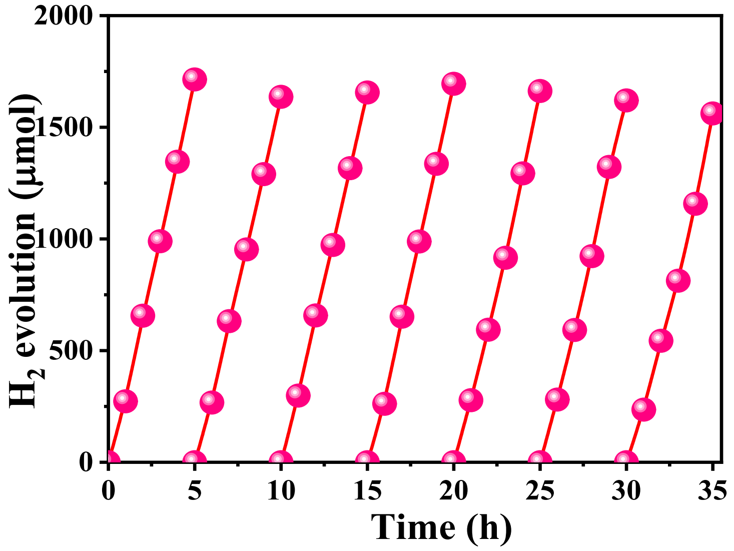

Fig. S33. Plot showing longer-term photocatalytic hydrogen evolution in pure water for TpPaCl under visible light irradiation. Reaction conditions: 5 mg TpPaCl, λ > 420 nm, 0.1 M ascorbic acid, hexachloroplatinic acid aqueous solution (2 wt% Pt to TpPaCl), room temperature, 35 h.

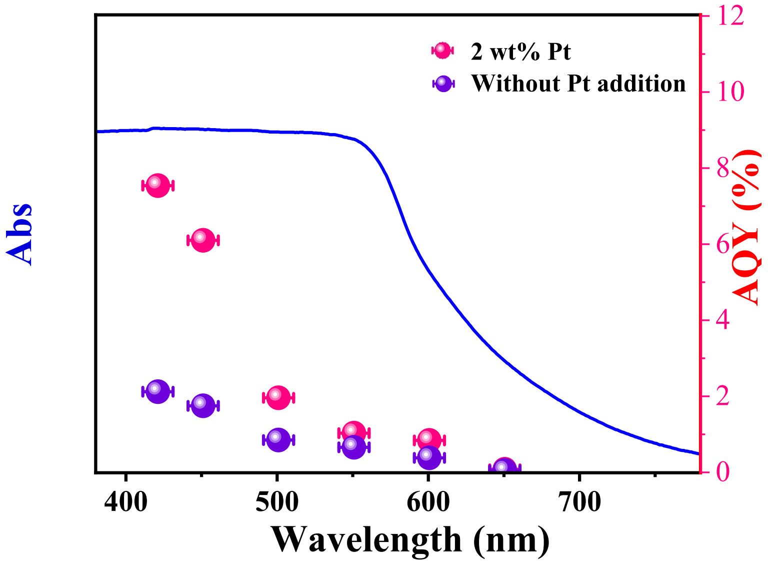

Fig. S34. Wavelength dependent AQE values of TpPaCl in pure water (pink) and seawater (purple). An Xe-lamp equipped with a series of band-pass flitters (420, 450, 500, 550, 600, and 650 nm) was used as the light source. Reaction conditions: 5 mg TpPaCl, 0.1 M ascorbic acid, hexachloroplatinic acid aqueous solution (2 wt% Pt to TpPaCl), room temperature, 5 h.

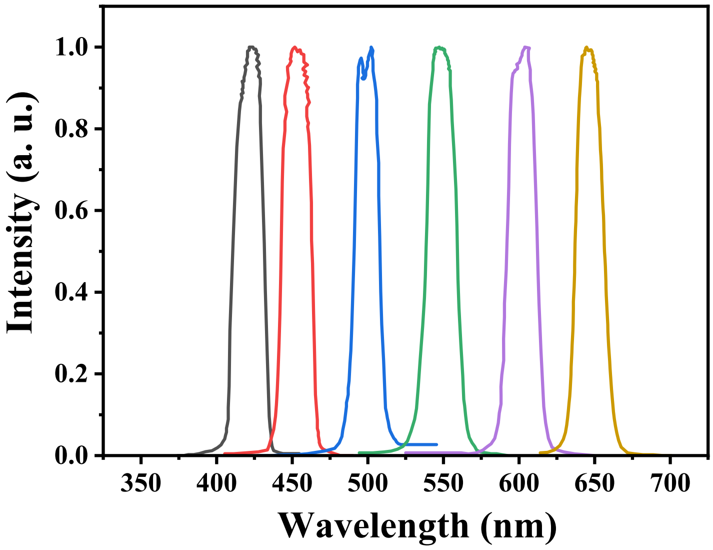

Fig. S35. UV-vis spectra of the filters (420, 450, 500, 550, 600, and 650 nm).

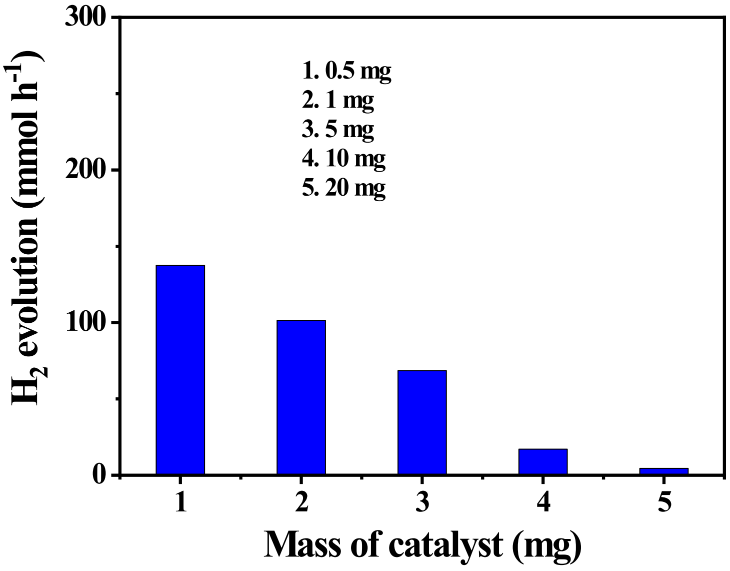

Fig. S36. Photocatalytic H_2 evolution over TpPaCl in pure water by using different catalyst dosages. Reaction conditions: λ > 420 nm, 0.1 M ascorbic acid, hexachloroplatinic acid aqueous solution (2 wt% Pt relative to TpPaCl), room temperature, 5 h.

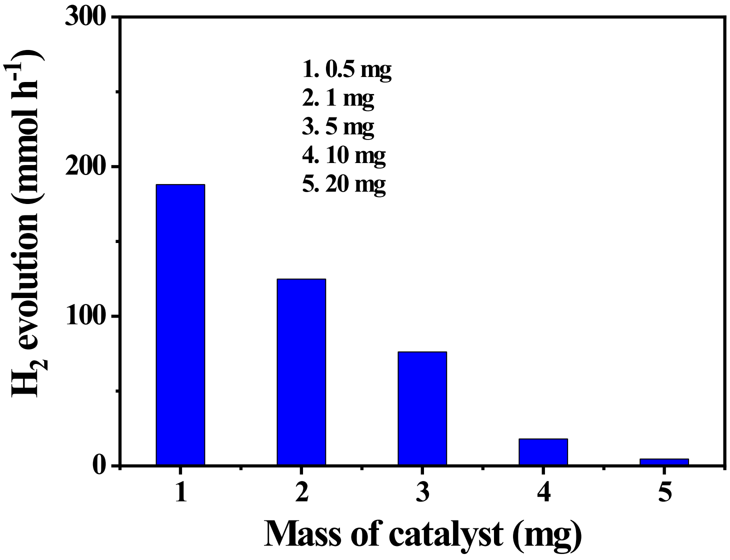

Fig. S37. Photocatalytic H_2 evolution over TpPaCl in seawater by using different catalyst dosages. Reaction conditions: $\lambda > 420$ nm, 0.1 M ascorbic acid, hexachloroplatinic acid aqueous solution (2 wt% Pt relative to TpPaCl), room temperature, 5 h.

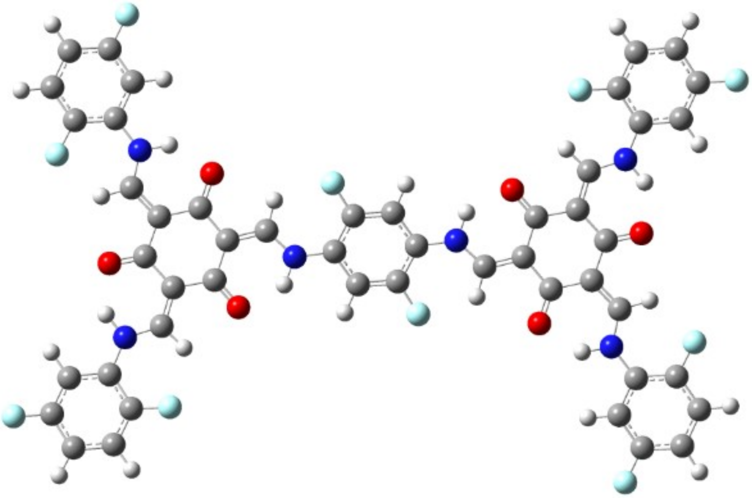

Fig. S38. Optimized geometrical structure of the representative fragment of TpPaF.

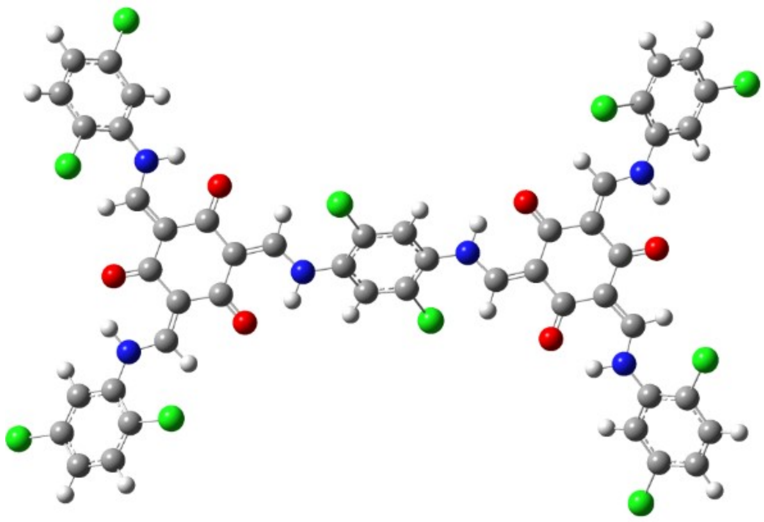

Fig. S39. Optimized geometrical structure of the representative fragment of TpPaCl.

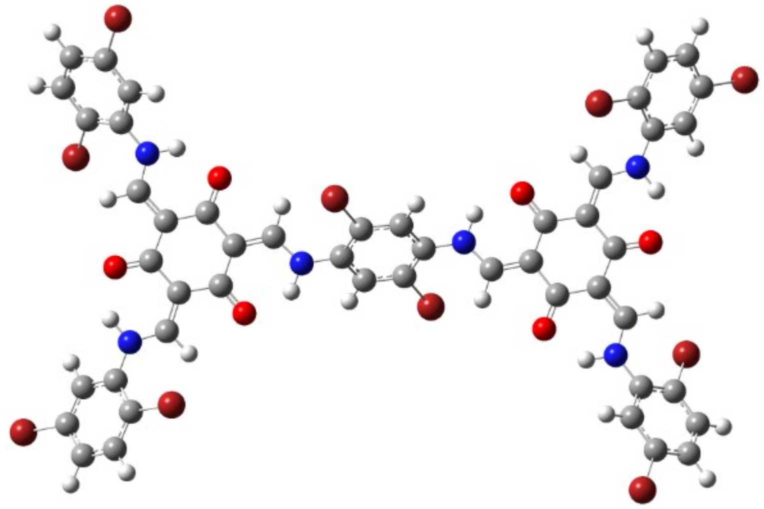

Fig. S40. Optimized geometrical structure of the representative fragment of TpPaBr.

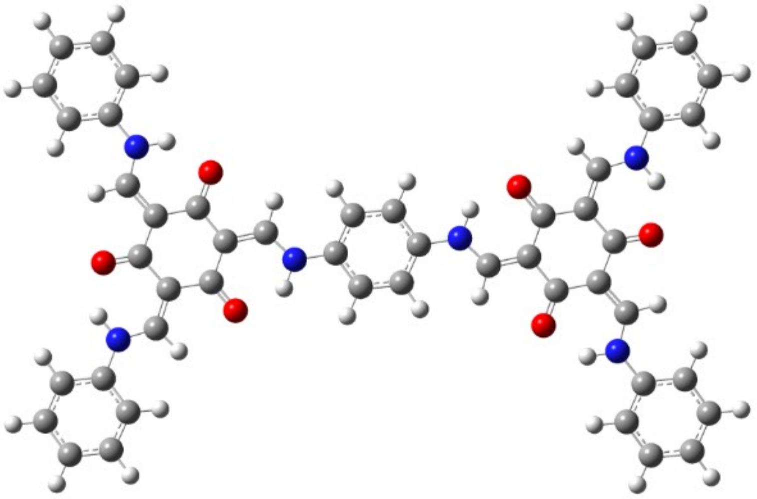

Fig. S41. Optimized geometrical structure of the representative fragment of TpPaH.

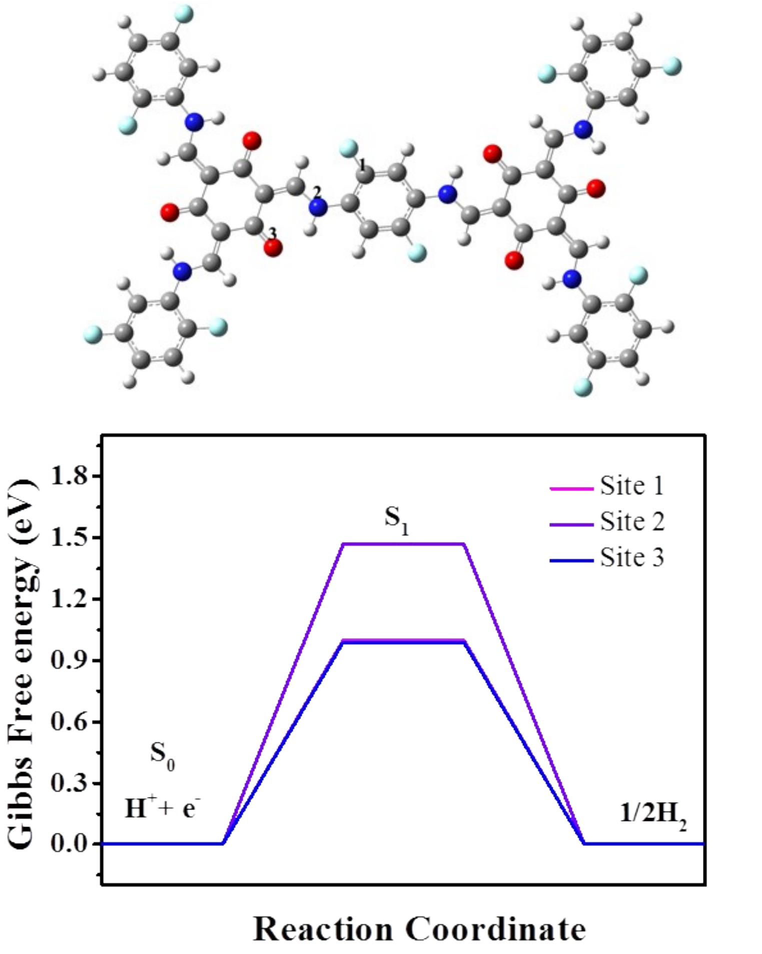

Fig. S42. The calculated free energies (eV) at different active sites of HER on TpPaF *via* the single-site process. Grey balls: C; white balls: H; red balls: O; blue balls: N; Indigo balls: F.

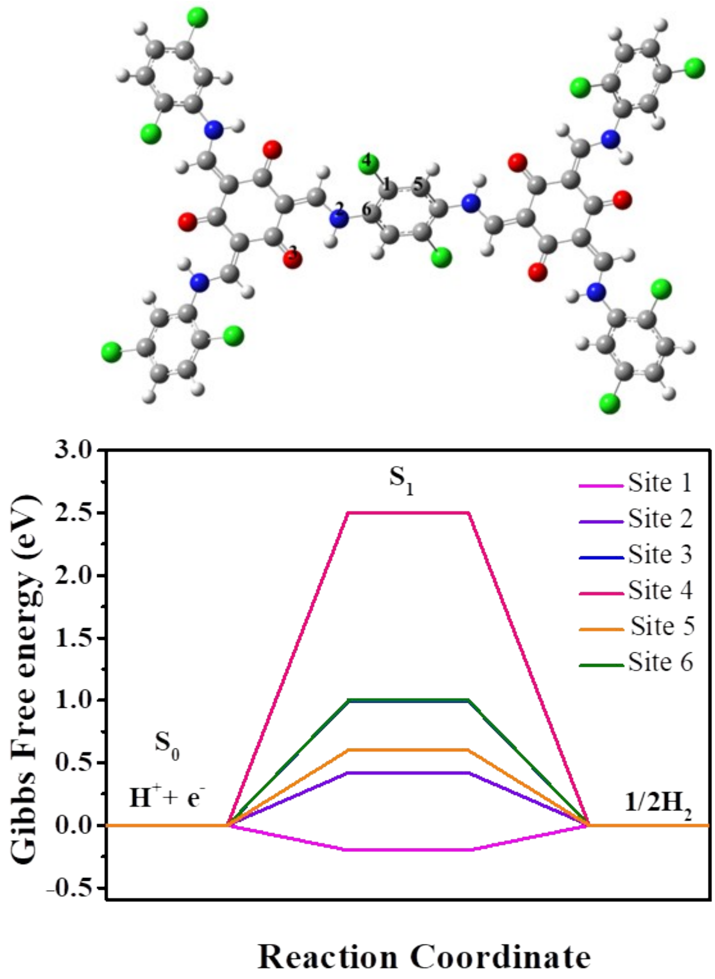

Fig. S43. The calculated free energies (eV) at different active sites of HER on TpPaCl *via* the single-site process. Grey balls: C; white balls: H; red balls: O; blue balls: N; green balls: Cl.

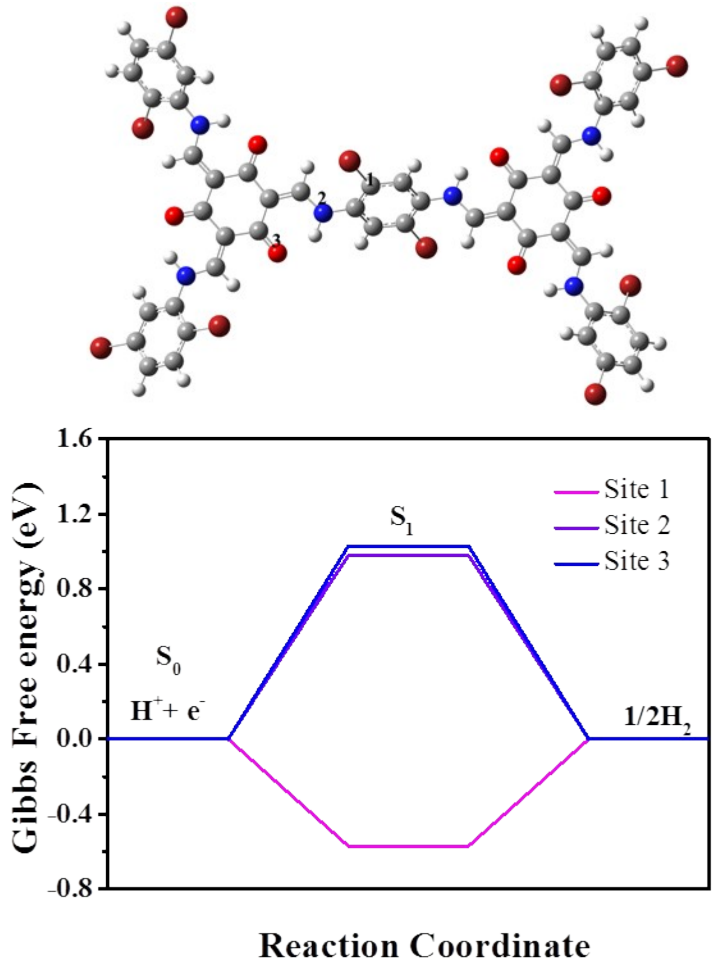

Fig. S44. The calculated free energies (eV) at different active sites of HER on TpPaBr *via* the single-site process. Grey balls: C; white balls: H; red balls: O; blue balls: N; maroon balls: Br.

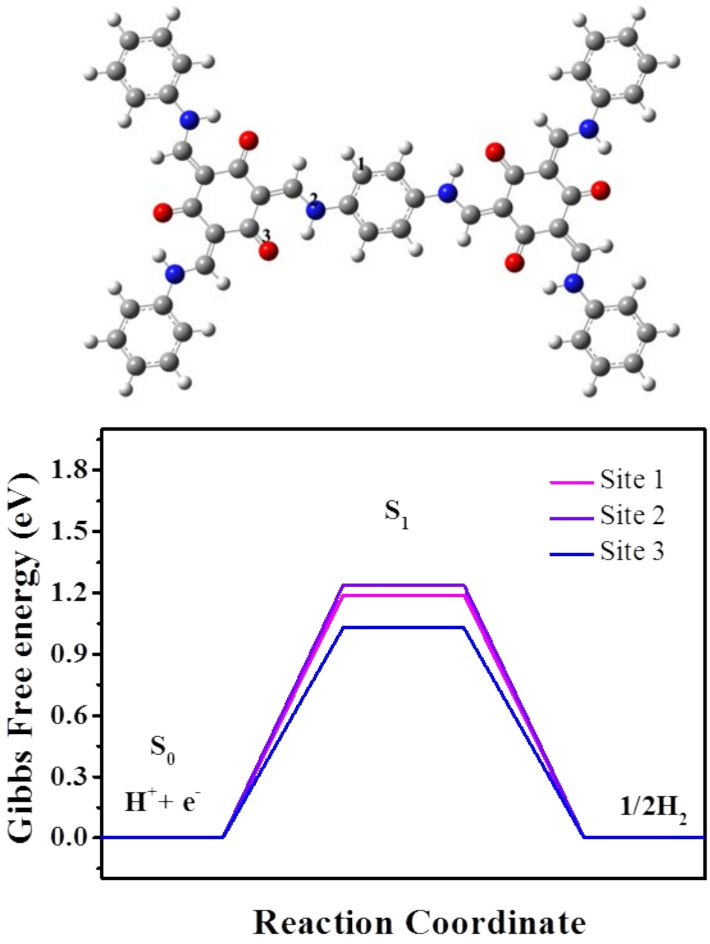

Fig. S45. The calculated free energies (eV) at different active sites of HER on TpPaH *via* the single-site process. Grey balls: C; white balls: H; red balls: O; blue balls: N.

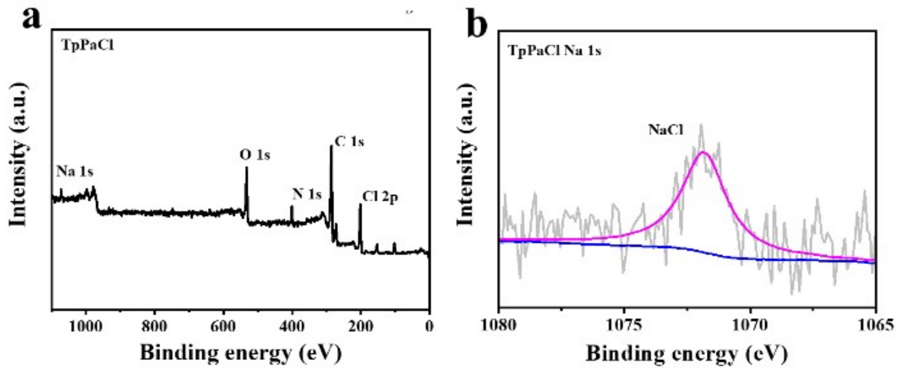

Fig. S46. (a) Survey scan XPS spectra of TpPaCl after adsorption of NaCl. High resolution (b) Na 1s XPS spectra of the TpPaCl after adsorption of NaCl.

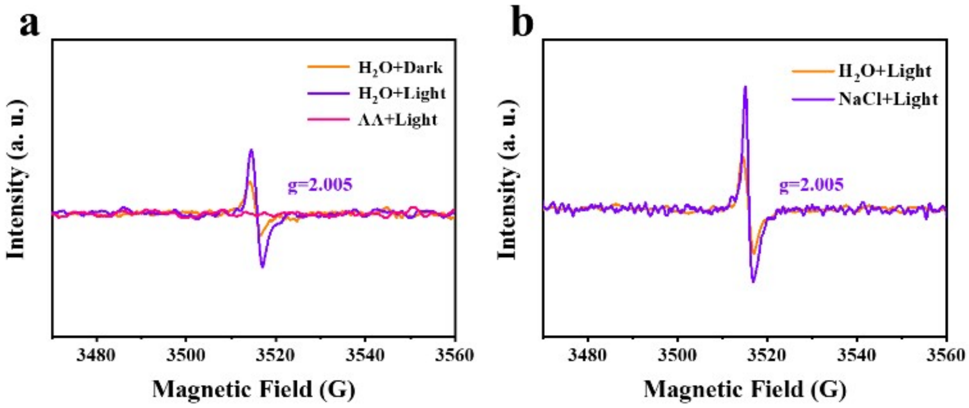

Fig. S47. (a) ESR signal of TpPaCl in H_2O under darkness, H_2O under visible light illumination, and 0.1 M AA solution under visible light illumination, respectively. (b) ESR signal of TpPaCl in H_2O and 3.5 wt% NaCl solution under visible light illumination.

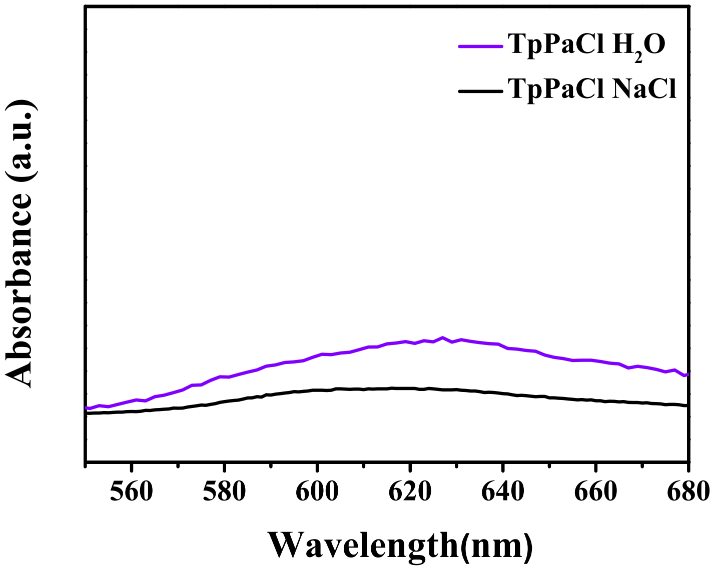

Fig. S48. Steady-state photoluminescence (PL) spectra of TpPaCl in H_2O and aqueous NaCl solution, respectively.

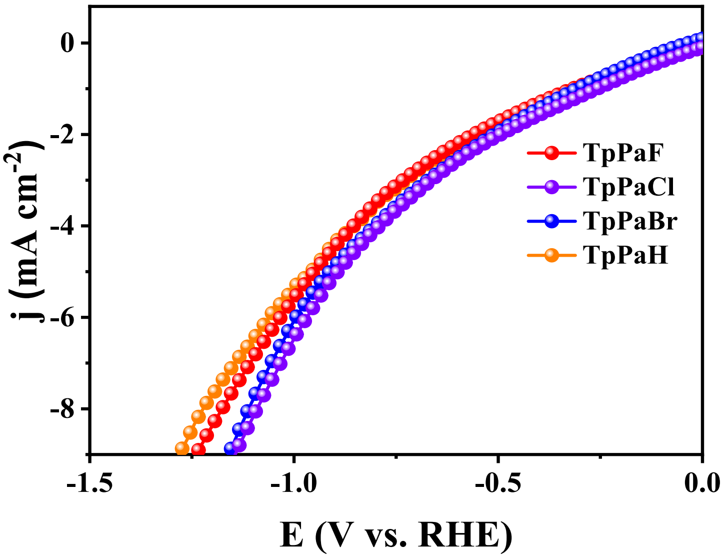

Fig. S49. HER polarization curves of TpPaX (X=F, Cl, Br, H).

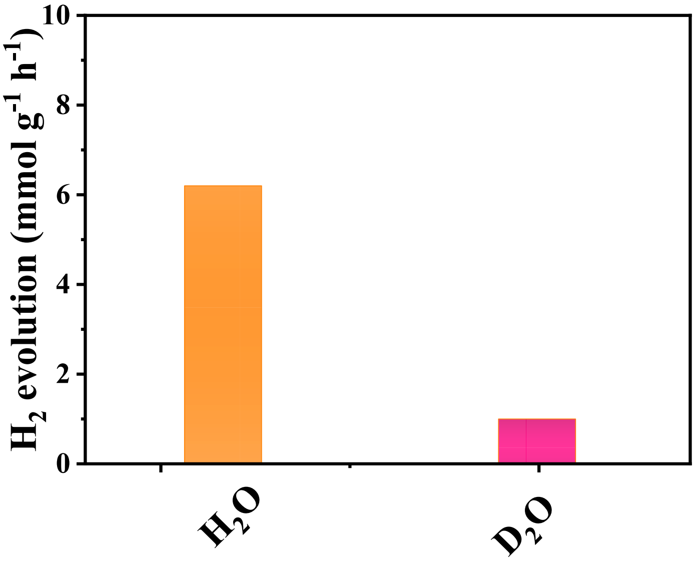

Fig. S50. Kinetic isotopic effects of HER over TpPaCl in H_2O and D_2O. Reaction conditions: 2 mg TpPaCl, λ > 420 nm, 0.1 M ascorbic acid, room temperature, 5 h.

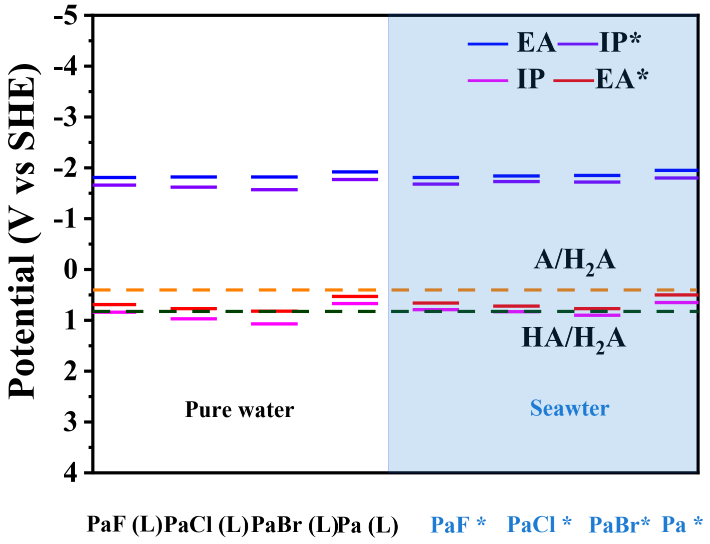

Fig. S51. (TD-)DFT calculation predicted ionization potential (IP), electron affinity (EA), and excited states (IP* and EA*) on the cut-out cluster models in pure water and seawater.

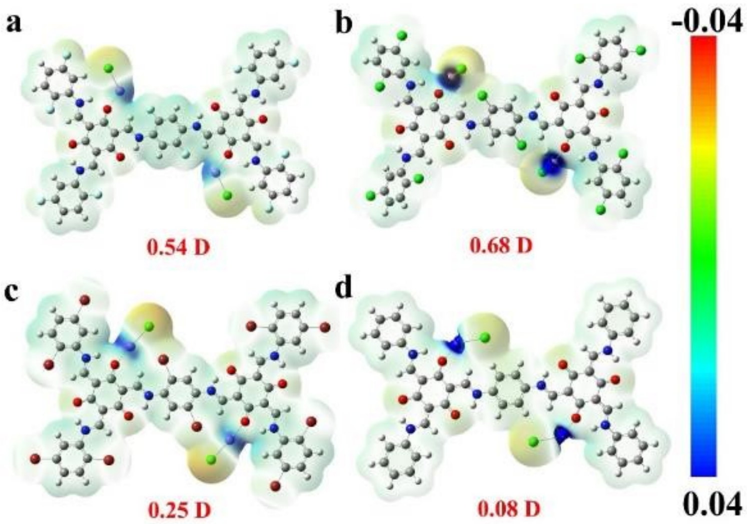

Fig. S52. The electrostatic potential distribution of (a) TpPaF, (b) TpPaCl, (c) TpPaBr, and (d) TpPaH in sweater.

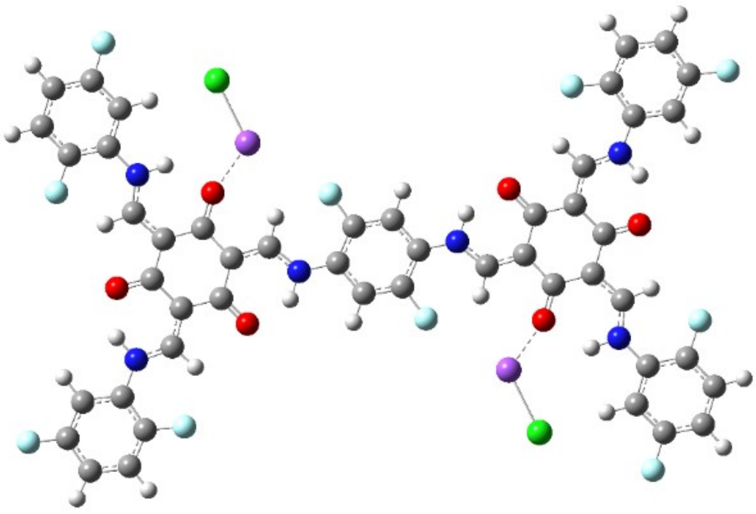

Fig. S53. Optimized geometrical structure of the representative fragment of TpPaF-NaCl.

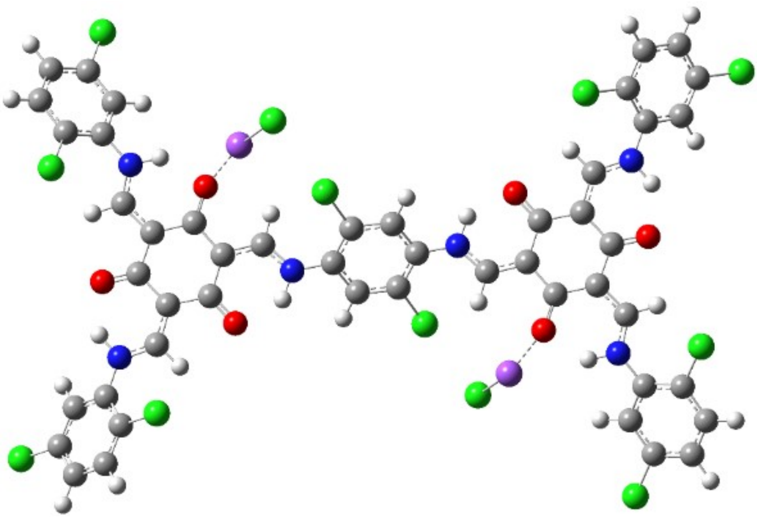

Fig. S54. Optimized geometrical structure of the representative fragment of TpPaCl-NaCl.

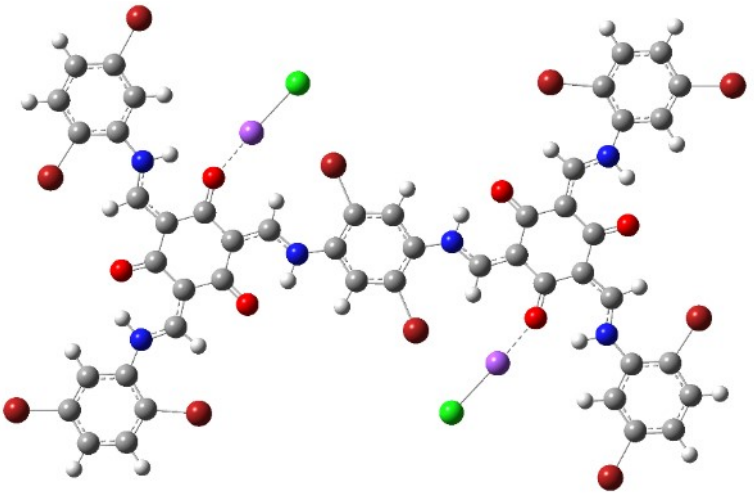

Fig. S55. Optimized geometrical structure of the representative fragment of TpPaBr-NaCl.

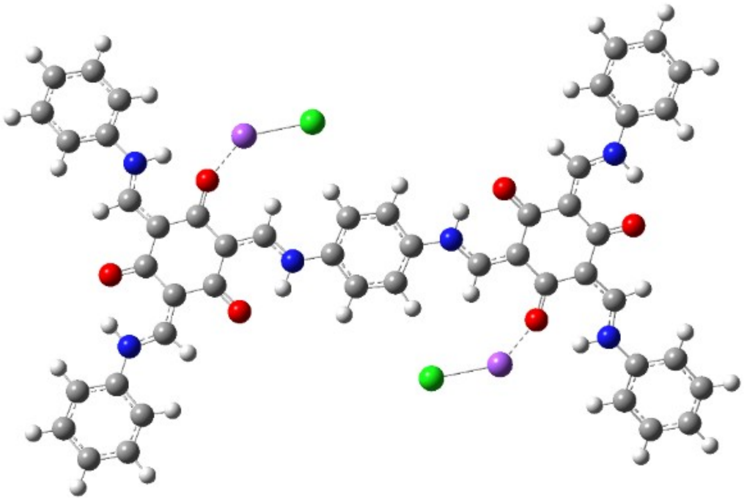

Fig. S56. Optimized geometrical structure of the representative fragment of TpPaH-NaCl.

Table S1. ICP-MS analysis

Sample	Entry	Pd (wt.%)	Pt (wt.%)	Au (wt.%)
	1	0.00005	0.00001	0.00002
**TpPaCl**	2	0.00005	0.00001	0.00002
	3	0.00005	0.00001	0.00002

Table S2. Fractional atomic coordination for the Pawley-refined TpPaF

TpPaF: Space group symmetry *P*6/M<br>a = b = 22.86 Å, c = 3.47 Å<br>α = β = 90°, γ = 120°							
Atom	x (Å)	y (Å)	z (Å)	Atom	x (Å)	y (Å)	z (Å)
C1	0.543487	-0.026157	0	N1	0.550017	0.141453	0
C2	0.570063	0.043717	0	N2	-0.141453	0.408563	0
C3	0.526233	0.070397	0	N3	-0.408563	-0.550017	0
C4	0.69284	0.407025	0	N4	-0.550017	-0.141453	0
C5	0.618707	0.359463	0	N5	0.141453	-0.408563	0
C6	0.807103	0.426637	0	N6	0.408563	0.550017	0
C7	0.026157	0.569643	0	O1	0.753045	0.285095	0
C8	-0.043717	0.526347	0	O2	-0.285095	0.46795	0
C9	-0.070397	0.455837	0	O3	-0.46795	-0.753045	0
C10	-0.407025	0.285815	0	O4	-0.753045	-0.285095	0
C11	-0.359463	0.259243	0	O5	0.285095	-0.46795	0
C12	-0.426637	0.380467	0	O6	0.46795	0.753045	0
C13	-0.569643	-0.543487	0	F1	0.638107	0.084263	0
C14	-0.526347	-0.570063	0	F2	-0.08426	0.553843	0
C15	-0.455837	-0.526233	0	F3	-0.55384	-0.63811	0
C16	-0.285815	-0.69284	0	F4	-0.63811	-0.08426	0
C17	-0.259243	-0.618707	0	F5	0.084263	-0.55384	0
C18	-0.380467	-0.807103	0	F6	0.553843	0.638107	0
C19	-0.543487	0.026157	0	H1	0.579583	-0.046953	0
C20	-0.570063	-0.043717	0	H2	0.824933	0.482967	0
C21	-0.526233	-0.070397	0	H3	0.512268	0.158425	0
C22	-0.69284	-0.407025	0	H4	0.046953	0.626537	0
C23	-0.618707	-0.359463	0	H5	-0.482967	0.341967	0
C24	-0.807103	-0.426637	0	H6	-0.158425	0.353843	0
C25	-0.026157	-0.569643	0	H7	-0.626537	-0.579583	0
C26	0.043717	-0.526347	0	H8	-0.341967	-0.824933	0
C27	0.070397	-0.455837	0	H9	-0.353843	-0.512268	0
C28	0.407025	-0.285815	0	H10	-0.579583	0.046953	0
C29	0.359463	-0.259243	0	H11	-0.824933	-0.482967	0
C30	0.426637	-0.380467	0	H12	-0.512268	-0.158425	0
C31	0.569643	0.543487	0	H13	-0.046953	-0.626537	0
C32	0.526347	0.570063	0	H14	0.482967	-0.341967	0
C33	0.455837	0.526233	0	H15	0.158425	-0.353843	0
C34	0.285815	0.69284	0	H16	0.626537	0.579583	0
C35	0.259243	0.618707	0	H17	0.341967	0.824933	0
C36	0.380467	0.807103	0	H18	0.353843	0.512268	0

Table S3. Fractional atomic coordination for the Pawley-refined TpPaCl

TpPaCl: Space group symmetry *P*6/M<br>a = b = 22.95 Å, c = 3.51 Å<br>α = β = 90°, γ = 120°							
Atom	x (Å)	y (Å)	z (Å)	Atom	x (Å)	y (Å)	z (Å)
C1	0.540887	-0.028497	0	N1	0.553433	0.141797	0
C2	0.570393	0.041457	0	N2	-0.141797	0.411637	0
C3	0.528827	0.070723	0	N3	-0.411637	-0.553433	0
C4	0.691443	0.406517	0	N4	-0.553433	-0.141797	0
C5	0.617827	0.358053	0	N5	0.141797	-0.411637	0
C6	0.806025	0.42827	0	N6	0.411637	0.553433	0
C7	0.028497	0.569383	0	O1	0.754673	0.287647	0
C8	-0.041457	0.528937	0	O2	-0.287647	0.467027	0
C9	-0.070723	0.458103	0	O3	-0.467027	-0.754673	0
C10	-0.406517	0.284927	0	O4	-0.754673	-0.287647	0
C11	-0.358053	0.259773	0	O5	0.287647	-0.467027	0
C12	-0.42827	0.377755	0	O6	0.467027	0.754673	0
C13	-0.569383	-0.540887	0	Cl1	0.658733	0.087557	0
C14	-0.528937	-0.570393	0	Cl2	-0.087557	0.571177	0
C15	-0.458103	-0.528827	0	Cl3	-0.571177	-0.658733	0
C16	-0.284927	-0.691443	0	Cl4	-0.658733	-0.087557	0
C17	-0.259773	-0.617827	0	Cl5	0.087557	-0.571177	0
C18	-0.377755	-0.806025	0	Cl6	0.571177	0.658733	0
C19	-0.540887	0.028497	0	H1	0.575317	-0.050967	0
C20	-0.570393	-0.041457	0	H2	0.822763	0.484157	0
C21	-0.528827	-0.070723	0	H3	0.515933	0.1588	0
C22	-0.691443	-0.406517	0	H4	0.050967	0.626283	0
C23	-0.617827	-0.358053	0	H5	-0.484157	0.338607	0
C24	-0.806025	-0.42827	0	H6	-0.1588	0.357133	0
C25	-0.028497	-0.569383	0	H7	-0.626283	-0.575317	0
C26	0.041457	-0.528937	0	H8	-0.338607	-0.822763	0
C27	0.070723	-0.458103	0	H9	-0.357133	-0.515933	0
C28	0.406517	-0.284927	0	H10	-0.575317	0.050967	0
C29	0.358053	-0.259773	0	H11	-0.822763	-0.484157	0
C30	0.42827	-0.377755	0	H12	-0.515933	-0.1588	0
C31	0.569383	0.540887	0	H13	-0.050967	-0.626283	0
C32	0.528937	0.570393	0	H14	0.484157	-0.338607	0
C33	0.458103	0.528827	0	H15	0.1588	-0.357133	0
C34	0.284927	0.691443	0	H16	0.626283	0.575317	0
C35	0.259773	0.617827	0	H17	0.338607	0.822763	0
C36	0.377755	0.806025	0	H18	0.357133	0.515933	0

Table S4. Fractional atomic coordination for the Pawley-refined TpPaBr

TpPaBr: Space group symmetry *P*6/M<br>a = b = 25.26 Å, c = 3.56 Å<br>α = β = 90°, γ = 120°							
Atom	x (Å)	y (Å)	z (Å)	Atom	x (Å)	y (Å)	z (Å)
C1	0.544198	0.977417	0	N1	0.556818	0.142702	0
C2	0.57389	0.041724	0	N2	-0.1427	0.414116	0
C3	0.532057	0.071172	0	N3	-0.41412	-0.55682	0
C4	0.695662	0.409015	0	N4	-0.55682	-0.1427	0
C5	0.621577	0.360241	0	N5	0.142702	-0.41412	0
C6	0.810979	0.430912	0	N6	0.414116	0.556818	0
C7	-0.97742	-0.43322	0	O1	0.759301	0.289391	0
C8	-0.04172	0.532166	0	O2	-0.28939	0.46991	0
C9	-0.07117	0.460885	0	O3	-0.46991	-0.7593	0
C10	-0.40902	0.286647	0	O4	-0.7593	-0.28939	0
C11	-0.36024	0.261336	0	O5	0.289391	-0.46991	0
C12	-0.43091	0.380067	0	O6	0.46991	0.759301	0
C13	0.433219	-0.5442	0	Br1	0.662791	0.088121	0
C14	-0.53217	-0.57389	0	Br2	-0.08812	0.57467	0
C15	-0.46089	-0.53206	0	Br3	-0.57467	-0.66279	0
C16	-0.28665	-0.69566	0	Br4	-0.66279	-0.08812	0
C17	-0.26134	-0.62158	0	Br5	0.088121	-0.57467	0
C18	-0.38007	-0.81098	0	Br6	0.57467	0.662791	0
C19	-0.5442	-0.97742	0	H1	0.57864	0.957806	0
C20	-0.57389	-0.04172	0	H2	0.532057	0.071172	0
C21	-0.53206	-0.07117	0	H3	0.825498	0.483867	0
C22	-0.69566	-0.40902	0	H4	0.522515	0.160864	0
C23	-0.62158	-0.36024	0	H5	-0.95781	-0.37917	0
C24	-0.81098	-0.43091	0	H6	-0.07117	0.460885	0
C25	0.977417	0.433219	0	H7	-0.48387	0.34163	0
C26	0.041724	-0.53217	0	H8	-0.16086	0.361651	0
C27	0.071172	-0.46089	0	H9	0.379166	-0.57864	0
C28	0.409015	-0.28665	0	H10	-0.46089	-0.53206	0
C29	0.360241	-0.26134	0	H11	-0.34163	-0.8255	0
C30	0.430912	-0.38007	0	H12	-0.36165	-0.52252	0
C31	-0.43322	0.544198	0	H13	-0.57864	-0.95781	0
C32	0.532166	0.57389	0	H14	-0.53206	-0.07117	0
C33	0.460885	0.532057	0	H15	-0.8255	-0.48387	0
C34	0.286647	0.695662	0	H16	-0.52252	-0.16086	0
C35	0.261336	0.621577	0	H17	0.957806	0.379166	0
C36	0.380067	0.810979	0	H18	0.071172	-0.46089	0

Table S5. Fractional atomic coordination for the Pawley-refined TpPaH

TpPaH: Space group symmetry *P*6/M<br>a = b = 22.76 Å, c = 3.49 Å<br>α = β = 90°, γ = 120°							
Atom	x (Å)	y (Å)	z (Å)	Atom	x (Å)	y (Å)	z (Å)
C1	0.546035	-0.023708	0	C34	0.286675	0.694267	0
C2	0.569683	0.046	0	C35	0.258662	0.619567	0
C3	0.523645	0.070002	0	C36	0.383217	0.808257	0
C4	0.694267	0.407592	0	N1	0.546557	0.141015	0
C5	0.619567	0.360905	0	N2	-0.141015	0.405542	0
C6	0.808257	0.42504	0	N3	-0.405542	-0.546557	0
C7	0.023708	0.569743	0	N4	-0.546557	-0.141015	0
C8	-0.046	0.523683	0	N5	0.141015	-0.405542	0
C9	-0.070002	0.453643	0	N6	0.405542	0.546557	0
C10	-0.407592	0.286675	0	O1	0.751457	0.28249	0
C11	-0.360905	0.258662	0	O2	-0.28249	0.468967	0
C12	-0.42504	0.383217	0	O3	-0.468967	-0.751457	0
C13	-0.569743	-0.546035	0	O4	-0.751457	-0.28249	0
C14	-0.523683	-0.569683	0	O5	0.28249	-0.468967	0
C15	-0.453643	-0.523645	0	O6	0.468967	0.751457	0
C16	-0.286675	-0.694267	0	H1	0.583792	-0.042785	0
C17	-0.258662	-0.619567	0	H2	0.626555	0.083583	0
C18	-0.383217	-0.808257	0	H3	0.827202	0.481847	0
C19	-0.546035	0.023708	0	H4	0.508502	0.157932	0
C20	-0.569683	-0.046	0	H5	0.042785	0.626577	0
C21	-0.523645	-0.070002	0	H6	-0.083583	0.542972	0
C22	-0.694267	-0.407592	0	H7	-0.481847	0.345355	0
C23	-0.619567	-0.360905	0	H8	-0.157932	0.35057	0
C24	-0.808257	-0.42504	0	H9	-0.626577	-0.583792	0
C25	-0.023708	-0.569743	0	H10	-0.542972	-0.626555	0
C26	0.046	-0.523683	0	H11	-0.345355	-0.827202	0
C27	0.070002	-0.453643	0	H12	-0.35057	-0.508502	0
C28	0.407592	-0.286675	0	H13	-0.583792	0.042785	0
C29	0.360905	-0.258662	0	H14	-0.626555	-0.083583	0
C30	0.42504	-0.383217	0	H15	-0.827202	-0.481847	0
C31	0.569743	0.546035	0	H16	-0.508502	-0.157932	0
C32	0.523683	0.569683	0	H17	-0.042785	-0.626577	0
C33	0.453643	0.523645	0	H18	0.083583	-0.542972	0

Table S6. Elemental analysis[a]

**Sample**		**C%**	**N%**	**H%**	**C/N**
**TpPaF**	Calcd.	59.27	8.64	2.69	6.86
	Found	49.68	8.36	4.02	5.94
**TpPaCl**	Calcd.	52.14	9.6	2.99	5.43
	Found	42.33	8.01	3.77	5.28
**TpPaBr**	Calcd.	39.96	7.36	2.29	5.43
	Found	33.96	6.19	2.94	5.49
**TpPaH**	Calcd.	68.25	12.57	4.82	5.43
	Found	54.12	10.03	4.22	5.40

[a]The slight difference between the measured chemical composition and the theoretical values is attributed to 1) the adsorption of water that decreased the content of C and N while increased the H content; 2) the inadequate condensation of monomers that caused the slight offset of the C/N ratio.

Table S7. Textural properties

Sample	S_{BET}[a] (m^2 g^{-1})	V_{total}[b] (cm^3 g^{-1})	D_{av}[c] (nm)
TpPaF	452	0.83	7.3
TpPaCl	493	0.54	4.4
TpPaBr	407	0.44	4.3
TpPaH	404	0.34	3.5

[a]Brunauer-Emmett-Teller (BET) surface area. [b]Total pore volume. [c]Average pore size.

Table S8. Summary of the fluorescence lifetime[a]

Sample	τ_1 (ns) (A_1)	τ_2 (ns) (A_2)	τ_{avg} (ns) (A)
TpPaF	0.68 (55.49%)	6.77 (44.51%)	3.4
TpPaCl	0.53 (23.09%)	20 (76.91%)	15.5
TpPaBr	0.60 (36.28%)	20 (63.72%)	13.0
TpPaH	0.51 (59.39%)	3.11 (40.61%)	1.6

[a]A_1+A_2=1.

Table S9. Hydrogen evolution performance without co-catalysts

Photocatalyst	Illumination	SED	Solution	Activity (μmol g^{-1} h^{-1})	AQE (%)	Ref.
TpPaCl	λ > 420 nm	AA	seawater	16300[a]	2.12 (420 nm)	This work
ZnPor-DETH-COF	λ > 420 nm	TEOA	pure water	403	0.063 (450 nm)	[2]
BT-COF	λ > 420 nm	AA	pure water	7700	7.53 (420 nm)	[3]
Py-ClTP-BT-COF	λ > 420 nm	AA	pure water	2200	N/A	[4]
FS-COF+WS5F	λ > 420 nm	AA	pure water	1300	N/A	[5]
CN/WP@NC	λ > 420 nm	TEOA	NaCl	1217	7.98 (420 nm)	[6]
CZS@S-1	λ > 420 nm	Na_2S/Na_2SO_3	$MgCl_2$	42000	N/A	[7]
$TiO_2.ZnS_2$	λ > 420 nm	Na_2S/Na_2SO_3	pure water	5503	N/A	[8]
$MoS_2@TiO_2$	AM 1.5	TEOA	pure water	21600	N/A	[9]
oCND-2	AM 1.5	TEOA	seawater	19700	N/A	[10]
$SiO_2/Ag@TiO_2$	λ > 400 nm	Glycerol	NaCl	800	N/A	[11]
CdxZn1-xSe	AM 1.5	Na_2SO_3 and Na_2S	seawater	36600	11.8 (520 nm)	[12]
Ti-O-Si (400)	λ > 420 nm	TEOA	NaCl	1640	N/A	[13]
$Ti_3C_2/Cd_{0.5}Zn_{0.5}S$	λ > 420 nm	Na_2SO_3 and Na_2S	seawater	9071	N/A	[14]
$WS_2@C-TiO_2$ on $g-C_3N_4$	λ > 420 nm	TEOA	seawater	1199	N/A	[15]
$COP-TF@CNi_2P$	λ > 420 nm	Na_2SO_3 and Na_2S	seawater	2500	N/A	[16]
UCOF-SCAU-2	λ > 420 nm	AA	pure water	96	6.87 (365 nm)	[17]
ZIS-P200	λ > 400 nm		seawater	92	0.16 (365 nm)	[18]

[a]2 mg COFs, λ > 420 nm, 300 mW/cm^2, 0.1 M ascorbic acid, pH=3.5, room temperature, 5 h.

Table S10. Hydrogen evolution performance with co-catalysts

Photocatalyst	Illumination	HEC	SED	Solution	Activity (μmol g^{-1} h^{-1})	AQE (%)	Ref.
TpPaCl	λ > 420 nm	Pt	AA	pure water	137631[a]	7.54 (420 nm)	This work
		Pt	AA	seawater	188122[b]		
ZnPor-DETH-COF	λ > 420 nm	Pt	TEOA	pure water	403	0.063 (450 nm)	[2]
BT-COF	λ > 420 nm	Pt	AA	pure water	7700	7.53 (420 nm)	[3]
Py-ClTP-BT-COF	λ > 420 nm	Pt	AA	pure water	8875	8.45 (420 nm)	[4]
FS-COF+WS5F	λ > 420 nm	Pt	AA	pure water	16300	3.2 (420 nm)	[5]
COF-JLU100	λ > 420 nm	Pt	TEOA	pure water	107308	5.13 (450 nm)	[19]
PY-DHBD-COF	λ > 420 nm	Pt	AA	pure water	71160	8.4 (450 nm)	[20]
COF-935	λ > 420 nm	Pt	AA	pure water	67550	3.39 (420 nm)	[21]
	λ > 420 nm	Pt	AA	seawater	18890		
TpPa	λ > 420 nm	Pt	AA	seawater	41300	0.57 (420 nm)	[22]
P-8CE	λ > 420 nm	Pt	TEA	seawater	38900	3.39 (420 nm)	[23]
COF-923	λ > 420 nm	Pt	AA	pure water	7824	0.68 (450 nm)	[24]
BUCT-COF-20	λ > 420 nm	Pt	AA	seawater	40360	N/A	[25]
COF-954	λ > 420 nm	Pt	AA	seawater	70410	N/A	[26]
TpPa(Δ)-Cu(II)-COF	λ > 420 nm	Cu	Cysteine(L)	pure water	14720	0.78 (600 nm)	[27]
Co/Zn-Salen-COF	λ > 420 nm	Co	AA	pure water	1378	0.78 (600 nm)	[28]
Pt-Tpy-COF	λ > 420 nm	Pt	AA	pure water	7800	3.39 (420 nm)	[29]

[a]0.5 mg COFs, λ > 420 nm, 300 mW/cm^2, 0.1 M ascorbic acid, pH=2.8, room temperature, 5 h. [b]0.5 mg COFs, λ > 420 nm, 300 mW/cm^2, 0.1 M ascorbic acid, 2 wt% Pt/TpPaCl, pH=3.5, room temperature, 5 h.

References

[1] S. Grimme, S. Ehrlich, L. Goerigk, Effect of the damping function in dispersion corrected density functional theory, *J., Comput. Chem.*, *2011*, **32**, 1456-1465.

[2] R. Chen, Y. Wang, Y. Ma, A. Mal, X.Y. Gao, L. Gao, L. Qiao, X.B. Li, L.Z. Wu, C. Wang, Rational design of isostructural 2D porphyrin-based covalent organic frameworks for tunable photocatalytic hydrogen evolution, *Nat. Commun.*, *2021*, **12**, 1354.

[3] T. Zhou, L. Wang, X. Huang, J. Unruangsri, H. Zhang, R. Wang, Q. Song, Q. Yang, W. Li, C. Wang, K. Takahashi, H. Xu, J. Guo, PEG-stabilized coaxial stacking of two-dimensional covalent organic frameworks for enhanced photocatalytic hydrogen evolution, *Nat. Commun.*, *2021*, **12**, 3934.

[4] W. Chen, L. Wang, D. Mo, F. He, Z. Wen, X. Wu, H. Xu, L. Chen, Modulating Benzothiadiazole-Based Covalent Organic Frameworks via Halogenation for Enhanced Photocatalytic Water Splitting, *Angew. Chem. Int. Ed.*, *2020*, **59**, 16902-16909.

[5] X. Wang, L. Chen, S.Y. Chong, M.A. Little, Y. Wu, W.H. Zhu, R. Clowes, Y. Yan, M.A. Zwijnenburg, R.S. Sprick, A.I. Cooper, Sulfone-containing covalent organic frameworks for photocatalytic hydrogen evolution from water, *Nat. Chem.*, *2018*, **10**, 1180-1189.

[6] T. Song, X. Zhang, K. Matras-Postolek, P. Yang, N-doped carbon layer promoted charge separation/transfer in WP/g-C3N4 heterostructures for efficient H_2 evolution and 4-nitrophenol removal, *Carbon*, *2023*, **202**, 378-388.

[7] Y. Yuan, F.-J. Wu, S.-T. Xiao, Y.-T. Wang, Z.-W. Yin, G. Van Tendeloo, G.-G. Chang, G. Tian, Z.-Y. Hu, S.-M. Wu, X.-Y. Yang, Hierarchical zeolites containing embedded $Cd_{0.2}Zn_{0.8}S$ as a photocatalyst for hydrogen production from seawater, *Chem. Commun.*, *2023*, **59**, 7275-7278.

[8] Q. Wang, G. Wang, J. Wang, J. Li, K. Wang, S. Zhou, Y. Su, In Situ Hydrothermal Synthesis of ZnS/TiO_2 Nanofibers S-Scheme Heterojunction for Enhanced Photocatalytic H_2 Evolution, *Adv. Sustainable Syst.*, *2023*, **7**, 2200027.

[9] X. Hu, S. Lu, J. Tian, N. Wei, X. Song, X. Wang, H. Cui, The selective deposition of MoS_2 nanosheets onto (101) facets of TiO_2 nanosheets with exposed (001) facets and their enhanced photocatalytic H_2 production, *Appl. Catal., B*, *2019*, **241**, 329-337.

[10] B. Jana, Y. Reva, T. Scharl, V. Strauss, A. Cadranel, D.M. Guldi, Carbon Nanodots for All-in-One Photocatalytic Hydrogen Generation, *J. Am. Chem. Soc.*, *2021*, **143**, 20122-20132.

[11] M. Gao, P.K.N. Connor, G.W. Ho, Plasmonic photothermic directed broadband sunlight harnessing for seawater catalysis and desalination, *Energy Environ. Sci.*, *2016*, **9**, 3151-3160.

[12] B. Qiu, Q. Zhu, M. Xing, J. Zhang, A robust and efficient catalyst of $Cd_xZn_{1-x}Se$ motivated by CoP for photocatalytic hydrogen evolution under sunlight irradiation, *Chem. Commun.*, *2017*, **53**, 897-900.

[13] T. Song, P. Zhang, T. Wang, A. Ali, H. Zeng, Constructing a novel strategy for controllable synthesis of corrosion resistant Ti^{3+} self-doped titanium–silicon materials with efficient hydrogen evolution activity from simulated seawater, *Nanoscale*, *2018*, **10**, 2275-2284.

[14] G. Zeng, Y. Cao, Y. Wu, H. Yuan, B. Zhang, Y. Wang, H. Zeng, S. Huang, $Cd_{0.5}Zn_{0.5}S/Ti_3C_2$ MXene as a Schottky catalyst for highly efficient photocatalytic hydrogen evolution in seawater, *Appl. Mater. Today*, *2021*, **22**, 100926.

[15] C. Yang, J. Qin, S. Rajendran, X. Zhang, R. Liu, WS_2 and C-TiO_2 Nanorods Acting as Effective Charge Separators on g-C3N4 to Boost Visible-Light Activated Hydrogen Production from Seawater, *ChemSusChem*, *2018*, **11**, 4077-4085.

[16] Y. Liu, Z. Xiang, Fully Conjugated Covalent Organic Polymer with Carbon-Encapsulated Ni_2P for Highly Sustained Photocatalytic H_2 Production from Seawater, *ACS Appl. Mater. Interfaces*, *2019*, **11**, 41313-41320.

[17] R. Shen, C. Qin, L. Hao, X. Li, P. Zhang, X. Li, Realizing Photocatalytic Overall Water Splitting by Modulating the Thickness-Induced Reaction Energy Barrier of Fluorenone-Based Covalent Organic Frameworks, *Adv. Mater.*, *2023*, **35**, 2305397.

[18] B.-J. Ng, W.-K. Chong, L.K. Putri, X.Y. Kong, J. Low, H.W. Lee, L.-L. Tan, W.S. Chang, S.-P. Chai, Heteroatom

P filling activates intrinsic S atomic sites of few-layered $ZnIn_2S_4$via modulation of H adsorption kinetics for sacrificial agent-free photocatalytic hydrogen evolution from pure water and seawater, *J. Mater. Chem. A*, *2023*, **11**, 17079-17090.
[19] S. Ma, T. Deng, Z. Li, Z. Zhang, J. Jia, Q. Li, G. Wu, H. Xia, S.W. Yang, X. Liu, Photocatalytic Hydrogen Production on a sp^2-Carbon-Linked Covalent Organic Framework, *Angew. Chem. Int. Ed.*, *2022*, **61**, e202208919.
[20] Y. Li, L. Yang, H. He, L. Sun, H. Wang, X. Fang, Y. Zhao, D. Zheng, Y. Qi, Z. Li, W. Deng, In situ photodeposition of platinum clusters on a covalent organic framework for photocatalytic hydrogen production, *Nat. Commun.*, *2022*, **13**, 1355.
[21] K. Wang, Y. Zhong, W. Dong, Y. Xiao, S. Ren, L. Li, Intermediate Formation of Macrocycles for Efficient Crystallization of 2D Covalent Organic Frameworks with Enhanced Photocatalytic Hydrogen Evolution, *Angew. Chem. Int. Ed.*, *2023*, **62**, e202304611.
[22] Q. Yue, G. Li, P. Fu, B. Meng, F. Ma, Y. Zhou, J. Wang, In-situ polarization of covalent organic frameworks in seawater enables enhanced photocatalytic hydrogen evolution under visible-light irradiation, *Nano Research*, *2023*, **16**, 6251-6259.
[23] C.L. Chang, T.F. Huang, W.C. Lin, L.Y. Ting, C.H. Shih, Y.H. Chen, J.J. Liu, Y.T. Lin, Y.T. Tseng, Y.H. Wu, Y.E. Sun, M.H. Elsayed, C.W. Chen, C.H. Yu, H.H. Chou, Synergistic Effect of Crown Ether and Main-Chain Engineering for Boosting Hydrogen Evolution of Polymer Photocatalysts in Seawater, *Adv. Energy Mater.*, *2023*, **13**, 2300986.
[24] W. Dong, Z. Qin, K. Wang, Y. Xiao, X. Liu, S. Ren, L. Li, Isomeric Oligo(Phenylenevinylene)-Based Covalent Organic Frameworks with Different Orientation of Imine Bonds and Distinct Photocatalytic Activities, *Angew. Chem. Int. Ed.*, *2022*, **62**, e202216073.
[25] Y. Wang, Z. Qiao, H. Li, R. Zhang, Z. Xiang, D. Cao, S. Wang, Molecular Engineering for Modulating Photocatalytic Hydrogen Evolution of Fully Conjugated 3D Covalent Organic Frameworks, *Angew. Chem. Int. Ed.*, *2024*, **63**, e202404726.
[26] Y. Zhong, W. Dong, S. Ren, L. Li, Oligo(phenylenevinylene)-Based Covalent Organic Frameworks with Kagome Lattice for Boosting Photocatalytic Hydrogen Evolution, *Adv. Mater.*, *2023*, **36**, 2308251.
[27] W. Weng, J. Guo, The effect of enantioselective chiral covalent organic frameworks and cysteine sacrificial donors on photocatalytic hydrogen evolution, *Nat. Commun.*, *2022*, **13**, 5768.
[28] W. Zhou, Q.W. Deng, H.J. He, L. Yang, T.Y. Liu, X. Wang, D.Y. Zheng, Z.B. Dai, L. Sun, C. Liu, H. Wu, Z. Li, W.Q. Deng, Heterogenization of Salen Metal Molecular Catalysts in Covalent Organic Frameworks for Photocatalytic Hydrogen Evolution, *Angew. Chem. Int. Ed.*, *2022*, **62**, e202214143.
[29] C. Liu, D.-L. Ma, P.-J. Tian, C. Jia, Q.-Y. Qi, G.-F. Jiang, X. Zhao, Lateral functionalization of a one-dimensional covalent organic framework for efficient photocatalytic hydrogen evolution from water, *J. Mater. Chem. A*, *2024*, **12**, 16063-16069.
